# Stem cell-derived exosomes for ischemic stroke: a conventional and network meta-analysis based on animal models

**DOI:** 10.3389/fphar.2024.1481617

**Published:** 2024-10-23

**Authors:** Kangli Xu, Xiaohui Zhao, Yuxuan He, Hongxin Guo, Yunke Zhang

**Affiliations:** ^1^ The First Affiliated Hospital of Henan University of Chinese Medicine, Zhengzhou, China; ^2^ The First Clinical Medical College of Henan University of Chinese Medicine, Zhengzhou, China

**Keywords:** stem cells, exosomes, stroke, animal experiments, animal models, meta-analysis

## Abstract

**Objective:**

We aimed to evaluate the efficacy of stem cell-derived exosomes for treating ischemic stroke and to screen for the optimal administration strategy.

**Methods:**

We searched PubMed, Web of Science, Embase, Cochrane Library, and Scopus databases for relevant studies published from their inception to 31 December 2023. Conventional and network meta-analyses of the routes of administration, types, and immune compatibility of stem cell-derived exosomes were performed using the cerebral infarct volume (%) and modified neurological severity score (mNSS) as outcome indicators.

**Results:**

A total of 38 randomized controlled animal experiments were included. Conventional meta-analysis showed that compared with the negative control group: intravenous administration significantly reduced the cerebral infarct volume (%) and mNSS; intranasal administration significantly reduced the cerebral infarct volume (%); and intracerebral administration significantly reduced the mNSS. Adipose-derived mesenchymal stem cell-derived exosomes (ADSC-Exos), bone marrow mesenchymal stem cell-derived exosomes (BMSC-Exos), dental pulp stem cell-derived exosomes (DPSC-Exos) and neural stem cell-derived exosomes (NSC-Exos) significantly reduced the cerebral infarct volume (%) and mNSS; Endothelial progenitor cell-derived exosomes (EPC-Exos), embryonic stem cell-derived exosomes (ESC-Exos), induced pluripotent stem cell-derived exosomes (iPSC-Exos) and neural progenitor cell-derived exosomes (NPC-Exos) significantly reduced the cerebral infarct volume (%); Umbilical cord mesenchymal stem cell-derived exosomes (UCMSC-Exos) significantly reduced the mNSS; and there was no significant difference between urogenital stem cell-derived exosomes (USC-Exos) and negative controls. Engineered modified exosomes had better efficacy than unmodified exosomes. Both allogeneic and xenogeneic stem cell-derived exosomes significantly reduced the cerebral infarct volume (%) and the mNSS. The network meta-analysis showed that intravenous administration was the best route of administration for reducing the cerebral infarct volume (%) and mNSS. Among the 10 types of stem cell-derived exosomes that were administered intravenously, BMSC-Exos were the best type for reducing the cerebral infarct volume (%) and the mNSS. Allogeneic exosomes had the best efficacy in reducing the cerebral infarct volume (%), whereas xenogeneic stem cell-derived exosomes had the best efficacy in reducing the mNSS.

**Conclusion:**

This meta-analysis, by integrating the available evidence, revealed that intravenous administration is the best route of administration, that BMSC-Exos are the best exosome type, that allogeneic exosomes have the best efficacy in reducing the cerebral infarct volume (%), and that xenogeneic exosomes have the best efficacy in reducing mNSS, which can provide options for preclinical studies. In the future, more high-quality randomized controlled animal experiments, especially direct comparative evidence, are needed to determine the optimal administration strategy for stem cell-derived exosomes for ischemic stroke.

**Systematic Review Registration:**

https://www.crd.york.ac.uk/PROSPERO/display_record.php?ID=CRD42024497333, PROSPERO, CRD42024497333

## 1 Introduction

Ischemic stroke is one of the major causes of death and disability among older adults worldwide. Research has shown that the number of ischemic stroke deaths worldwide has increased from 2.04 million to 3.29 million between 1990 and 2019, and is expected to increase further to 4.9 million by 2030 (Fan et al., 2023; Krishnamurthi et al., 2020). Early thrombolysis and thrombectomy are currently effective treatments for ischemic stroke (Kleindorfer et al., 2021). However, although thrombolysis and thrombectomy therapies promote recanalization of occluded cerebral arteries, neurological function is not fully restored in nearly 50% of stroke patients treated with revascularization therapy (Fischer et al., 2022). Many patients are not only subject to a strict time window, but after thrombolysis or thrombectomy, brain tissue is often not effectively repaired because of microcirculatory deficits such as ischemia/reperfusion injury, hemorrhagic transformation, and “no-reflow” (Elfil et al., 2023; Qiu et al., 2023). Hence, the exploration of new therapeutic strategies is urgently needed.

Stem cells, with their ability for self-renewal, differentiation, and tissue repair, are promising for treating ischemic stroke and have moved from the laboratory stage into early clinical trials (Ya et al., 2024; Yamaguchi et al., 2022). Studies have shown that transplanted stem cells release bioactive substances into the brain mainly through paracrine effects to reverse or repair the pathological damage caused by ischemia, in which exosomes play a key role (Zhou et al., 2019; Asgari Taei et al., 2022). Exosomes are capable of delivering substances such as proteins, lipids, nucleic acids, and cellular metabolites to recipient cells, thereby mediating cellular communication and regulating cellular function (Kalluri and LeBleu, 2020). Exosomes, as a type of cell-free therapy, not only inherit the biological functions of their source cells, but also avoid the safety concerns associated with cell-based therapies due to their low tumorigenicity, immunogenicity, and ability to cross the blood-brain barrier (Abdulmalek et al., 2024). Thus, stem cell-derived exosomes show great promise for treating stroke and other central nervous system diseases.

Increasingly, preclinical studies have demonstrated that stem cell-derived exosomes promote endogenous neural circuit remodeling, neurovascular neogenesis, and brain tissue repair, with significant efficacy in animal models of ischemic stroke (Dehghani et al., 2021; Zhang et al., 2019). Several narrative reviews have summarized the mechanisms and research progress of various stem cell-derived exosomes for treating ischemic stroke (Dehghani et al., 2021; Waseem et al., 2023; Xiong et al., 2022). However, differences in the stem cell source of exosomes, route of administration, and immune compatibility cause differences in efficacy. The optimal strategy for the administration of stem cell-derived exosomes for the treatment of ischemic stroke remains unclear. To date, few studies have directly compared the efficacy of different stem cell-derived exosome treatment strategies. In the absence of evidence for direct comparisons, network meta-analysis establishes indirect comparisons of treatment strategies and thus estimates and ranks the relative effectiveness of all interventions (Van Valkenhoef et al., 2012). Therefore, we performed conventional and network meta-analyses of the routes of administration, types, and immune compatibility of stem cell-derived exosomes, using the cerebral infarct volume (%) and mNSS as outcome indicators; and qualitative synthesis of factors that were too heterogeneous to be quantitatively analyzed. These findings will provide support and reference for improving the efficacy of stem cell-derived exosomes for the treatment of ischemic stroke and accelerating their clinical translation.

## 2 Materials and methods

This meta-analysis protocol followed the Preferred Reporting Items for Systematic Reviews and Meta-Analysis (PRISMA) 2020 Statement (Page et al., 2021). The protocol has been registered in PROSPERO (registration ID: CRD42024497333).

### 2.1 Search strategy

We comprehensively searched the PubMed, Web of Science, Embase, Cochrane Library, and Scopus databases for English-language studies from their inception until 31 December 2023. We searched for “ischemic stroke,” “stem cell,” and “exosome” as MeSH and free terms. The detailed search strategies for each database are documented in Supplementary Material S1.

### 2.2 Study inclusion and exclusion criteria

The inclusion and exclusion criteria were developed following the PICOS principles (Participants, Intervention, Comparison, Outcome, and Study). 1) Animals and diseases (P): Rat/mouse ischemic stroke models were included; concomitant comorbidities, other animal models, *in vitro* studies, and clinical studies were excluded. 2) Intervention (I): Studies in which stem cell-derived exosomes were used to treat ischemic stroke in rats/mice were included; non-stem cell-derived exosomal studies were excluded. 3) Comparison(C): Positive controls for comparison of different stem cell-derived exosomes and negative controls such as vector, PBS, and saline were included; studies without negative controls were excluded. 4) Outcome (O): Studies reporting at least one of the outcomes in terms of cerebral infarct volume (%), the mNSS; studies with statistically unclear descriptions of outcome metrics and those for which complete data were not available were excluded. 5) Study (S): Randomized controlled studies were included; case reports, and controlled studies before and after the same group of treatments were excluded. Other: Only English-language studies published before 31 December 2023, were included; publications such as reviews, pathology reports, conference abstracts, and letters were excluded.

### 2.3 Literature screening and data extraction

Two independent reviewers performed screening on the basis of pre-established inclusion and exclusion criteria. After duplicate publications were eliminated, titles and abstracts were assessed to exclude ineligible studies. The reviewers subsequently obtained full texts to screen the literature critically and identify studies for final inclusion. The extracted data included the following: 1) first author, year of publication, country; 2) animal characteristics (species, sex, age, weight); 3) grouping method, sample size (experimental/control group); 4) methods of modeling ischemic stroke; 5) exosome characterization (stem cell source, isolation and purification methods, particle size, and immune compatibility); 6) drug delivery strategy (route of administration, timing, dose, frequency, duration of treatment); and 7) outcome (mean, standard deviation or standard error). Disagreements were resolved by discussion with the corresponding author.

### 2.4 Outcome indicators

This meta-analysis used the cerebral infarct volume (%) and the mNSS as outcome indicators, both of which are continuous data. If assessments were performed at different times, only the results of the longest follow-up time were extracted. In addition, if one study performed a comparison of exosomes of different stem cell origins, they would need to be extracted as a separate dataset. If numerical data were not reported in the publication, Web Plot Digitizer 4.6 software was used to extract the mean, average, standard deviation, or standard error from the graphical data (Drevon et al., 2017). The formula for converting standard error to standard deviation is as follows: SD = SME×√n.

### 2.5 Quality assessment

Two independent reviewers used the Systematic Review Centre for Laboratory Animal Experimentation (SYRCLE)’s Risk of Bias Tool to assess potential bias in each of the included studies (Hooijmans et al., 2014). The tool contains 6 assessment domains, namely, selection bias, performance bias, detection bias, attrition bias, reporting bias, and other bias, with a total of 10 entries. Each entry was judged to be “low risk,” “high risk,” or “unclear,” and inconsistencies were discussed and resolved with the corresponding author.

### 2.6 Statistical analysis

Conventional meta-analysis: A conventional meta-analysis of the routes of administration, types, and immune compatibility of stem cell-derived exosomes was performed via Review Manager 5.3 software. The cerebral infarct volume (%) and the mNSS are continuous data, so they are presented as the means and standard deviations. The effect size of each outcome indicator was combined via the standardized mean difference (SMD) and its 95% confidence interval (CI), with *p* < 0.05 being significant. The animal experimental studies used a random effects model to combine effect sizes (Liu et al., 2024). *Cochran’s Q* test and *I* ^*2*^ were used to assess the heterogeneity of the included studies with *p* < 0.1 and *I* ^*2*^ > 50% suggesting significant heterogeneity.

Network meta-analysis: Bayesian random-effects network meta-analysis of the routes of administration, types, and immune compatibility of stem cell-derived exosomes was performed via ADDIS 1.16.8 software (Shi et al., 2022). First, a network evidence map was constructed for each outcome indicator, with each node in the map representing an intervention, the lines connecting the nodes representing direct comparisons between the two interventions, and the width of the lines representing the number of studies comparing the two interventions. Second, if there was a closed loop in the network evidence map, inconsistency between direct and indirect evidence was detected via node splitting, with *p* > 0.05 indicating that the results of direct and indirect comparisons between interventions were consistent, the consistency model was used; conversely, the inconsistency model was used. Third, effect sizes for each outcome indicator were combined via SMD, and the 95% CI and ranked probability rankings were plotted. Fourth, subgroup analyses of species were performed. Fifth, potential publication bias was assessed via funnel plots, Begg’s test, and Egger’s test, with *p* < 0.05 suggesting significant publication bias.

## 3 Results

### 3.1 Results of the search

**FIGURE 1:**
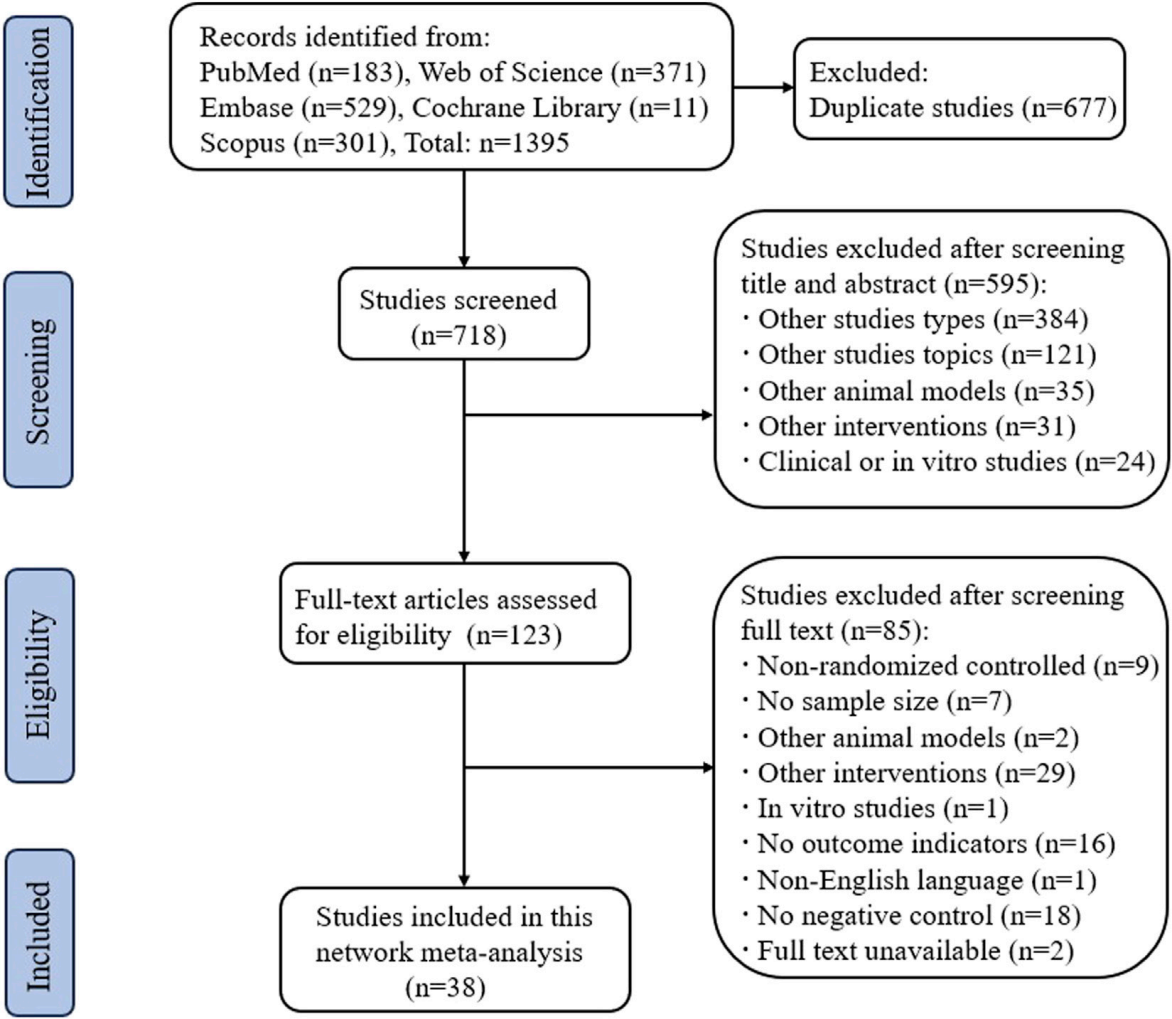
Flow chart of the literature selection process.

We searched a total of 1395 publications in the PubMed, Web of Science, Embase, Cochrane Library, and Scopus databases. After screening in strict accordance with the inclusion and exclusion criteria, 38 randomized controlled animal experiments were ultimately included, and the detailed screening process is shown in Figure 1.

### 3.2 Characteristics of the included studies

**FIGURE 2:**
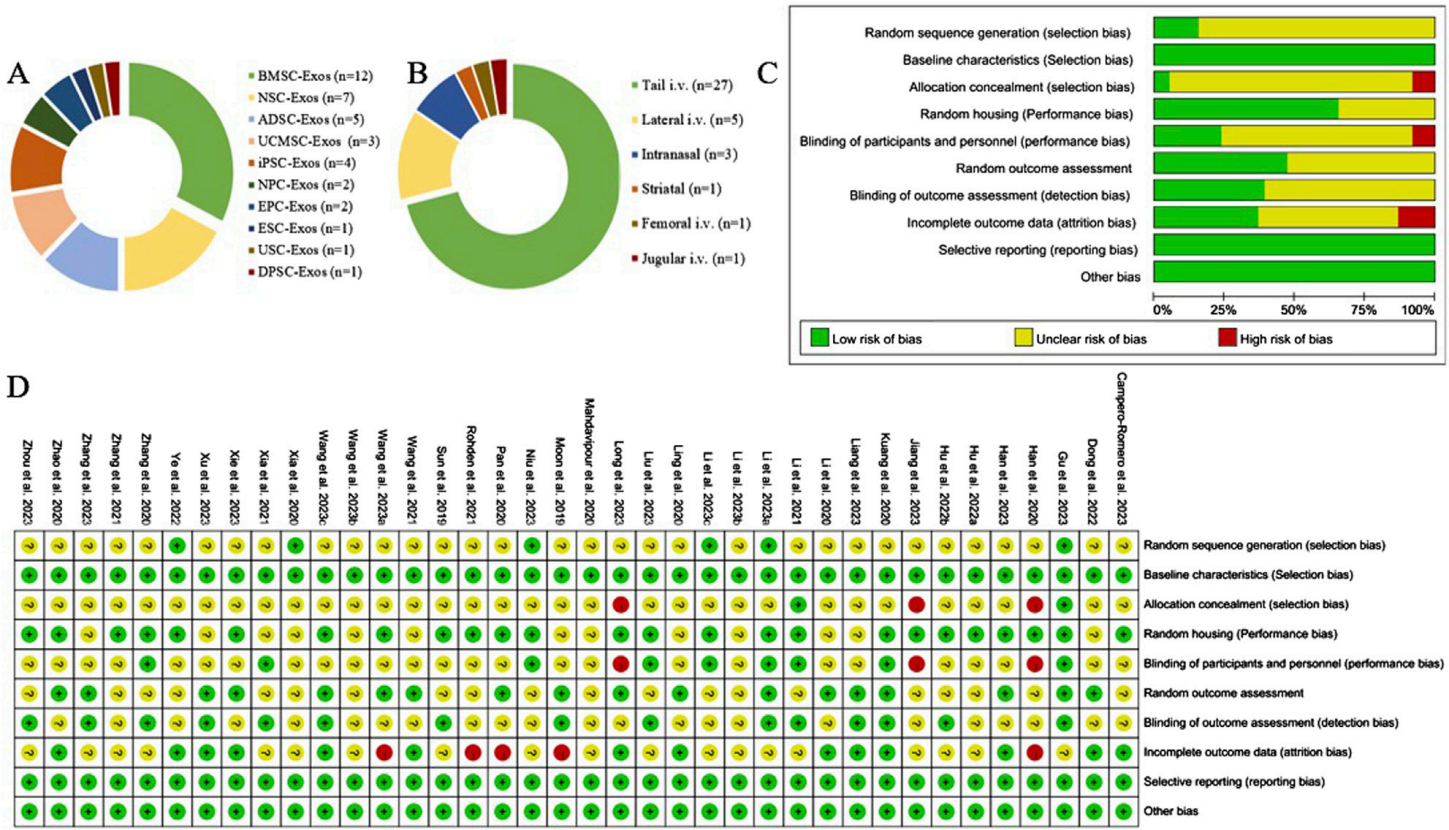
Exosome characterization and risk of bias for publications. **(A)** Types of stem cell-derived exosomes; **(B)** routes of administration of stem cell-derived exosomes; **(C)** risk of bias of the included studies; **(D)** summarized risk of bias of the included studies.

Among the 38 studies, 16 (Wang et al., 2023a; Long et al., 2023; Li Y. et al., 2023; Jiang et al., 2023; Ye et al., 2022; Dong et al., 2022; Wang et al., 2021; Rohden et al., 2021; Zhao et al., 2020; Zhang et al., 2020; Xia et al., 2020; Mahdavipour et al., 2020; Ling et al., 2020; Li et al., 2020; Han et al., 2020; Moon et al., 2019) were in rats, and 22 (Liang et al., 2024; Zhou et al., 2023; Zhang et al., 2023; Xu et al., 2023; Xie et al., 2023; Wang et al., 2023b; Wang J. et al., 2023; Niu et al., 2023; Liu et al., 2023; Li R. et al., 2023; Li Q. et al., 2023; Han et al., 2023; Gu et al., 2023; Campero-Romero et al., 2023; Hu X. et al., 2022; Hu H. et al., 2022; Zhang et al., 2021; Xia et al., 2021; Li et al., 2021; Pan et al., 2020; Kuang et al., 2020; Sun et al., 2019) were in mice. A total of 10 stem cell-derived exosomes were involved. Thirteen studies used bone marrow mesenchymal stem cell-derived exosomes (BMSC-Exos); seven studies used neural stem cell-derived exosomes (NSC-Exos); five studies used adipose-derived mesenchymal stem cell-derived exosomes (ADSC-Exos); four studies used induced pluripotent stem cell-derived exosomes (iPSC-Exos); three studies used umbilical cord mesenchymal stem cell-derived exosomes (UCMSC-Exos); two studies used neural progenitor cell-derived exosomes (NPC-Exos); two studies used endothelial progenitor cell-derived exosomes (EPC-Exos); one study used embryonic stem cell-derived exosomes (ESC-Exos); one study used urogenital stem cell-derived exosomes (USC-Exos); and one study used dental pulp stem cell-derived exosomes (DPSC-Exos). Most studies used differential ultracentrifugation to extract exosomes, five studies used exosome isolation and purification kits, two studies used polyethylene glycol precipitation combined with ultrafiltration, and one study used anion exchange. The particle size of the exosomes ranged from 30–200 nm, and 2 publications did not report the particle size of the exosomes. The majority of the studies used intravenous administration, six studies used intracerebral administration, and three studies used intranasal administration. A total of 17 studies used engineered modified exosomes, of which 10 studies endogenously loaded stem cell-derived exosomes with RNAs and non-coding RNAs, 5 studies extracted exosomes after pretreatment of stem cells in culture (including drug-containing serum, small-molecule actives, brain tissue extracts, and hydrogel 3D culture), and 2 studies surface-modified exosomes (RGD/Angiopep-2 peptide, hyaluronic acid hydrogel). The particle size of these engineered modified exosomes ranged from 30–200 nm. The characteristics of the included publications are shown in Supplementary Table S1 and Figures 2A,B, and detailed administration strategies for stem cell-derived exosomes are provided in Supplementary Table S2.

### 3.3 Quality assessment of the included studies

Of the 38 randomized controlled studies, 6 (15.79%) described the method of random sequence generation and were rated as “low risk”; the remaining studies mentioned only “randomized grouping” and did not describe the detailed method of random sequence generation and were rated as “unclear.” All the animal experiments ensured that there were no differences in baseline characteristics. Three (7.89%) studies had different routes of administration for each group and did not describe whether the investigators were blinded, therefore, these studies were rated as “high risk” for allocation concealment and performance bias. Nine (23.68%) studies were blinded to researchers and breeders and were rated as “low risk.” Fifteen studies were blinded to the outcome assessors and were rated as “low risk.” Fourteen (36.84%) of the studies provided details of lost visits and were rated as “low risk.” The results of the detailed risk of bias assessment are shown in Figures 2C,D.

### 3.4 Effect of stem cell-derived exosomes on the cerebral infarction volume (%)

A total of 29 studies (Wang et al., 2023a; Long et al., 2023; Li Y. et al., 2023; Jiang et al., 2023; Ye et al., 2022; Dong et al., 2022; Wang et al., 2021; Rohden et al., 2021; Xia et al., 2020; Ling et al., 2020; Han et al., 2020; Zhou et al., 2023; Zhang et al., 2023; Xu et al., 2023; Xie et al., 2023; Wang J. et al., 2023; Niu et al., 2023; Liu et al., 2023; Li R. et al., 2023; Li Q. et al., 2023; Han et al., 2023; Gu et al., 2023; Hu H. et al., 2022; Zhang et al., 2021; Xia et al., 2021; Li et al., 2021; Pan et al., 2020; Kuang et al., 2020; Sun et al., 2019) reported the cerebral infarct volume (%), which included 149 rats and 321 mice and included 10 types of exosomes (ADSC-Exos, BMSC-Exos, DPSC-Exos, EPC-Exos, ESC-Exos, iPSC-Exos, NPC-Exos, NSC-Exos, UCMSC-Exos, and USC-Exos), with 1 study (Xu et al., 2023) performing a direct comparison between NPC-Exos and EPC-Exos. Twenty-five studies used intravenous administration (23 via the tail vein, 1 via the femoral vein, and 1 via the jugular vein), 2 studies used intranasal administration, and 2 studies used intracerebral administration (lateral ventricular injection). Thirteen studies involved allogeneic exosomes, and 16 studies involved xenogeneic exosomes. A total of 11 studies used modified exosomes, with 5 studies endogenously loading non-coding RNAs, 1 study endogenously loading RNAs, 4 studies pretreating stem cells (including drug-containing serum, brain tissue extracts, and hydrogel three-dimensional cultures), and 1 study surface-modifying the exosomes using adherent hyaluronic acid hydrogels.

#### 3.4.1 Conventional meta-analysis

**FIGURE 3:**
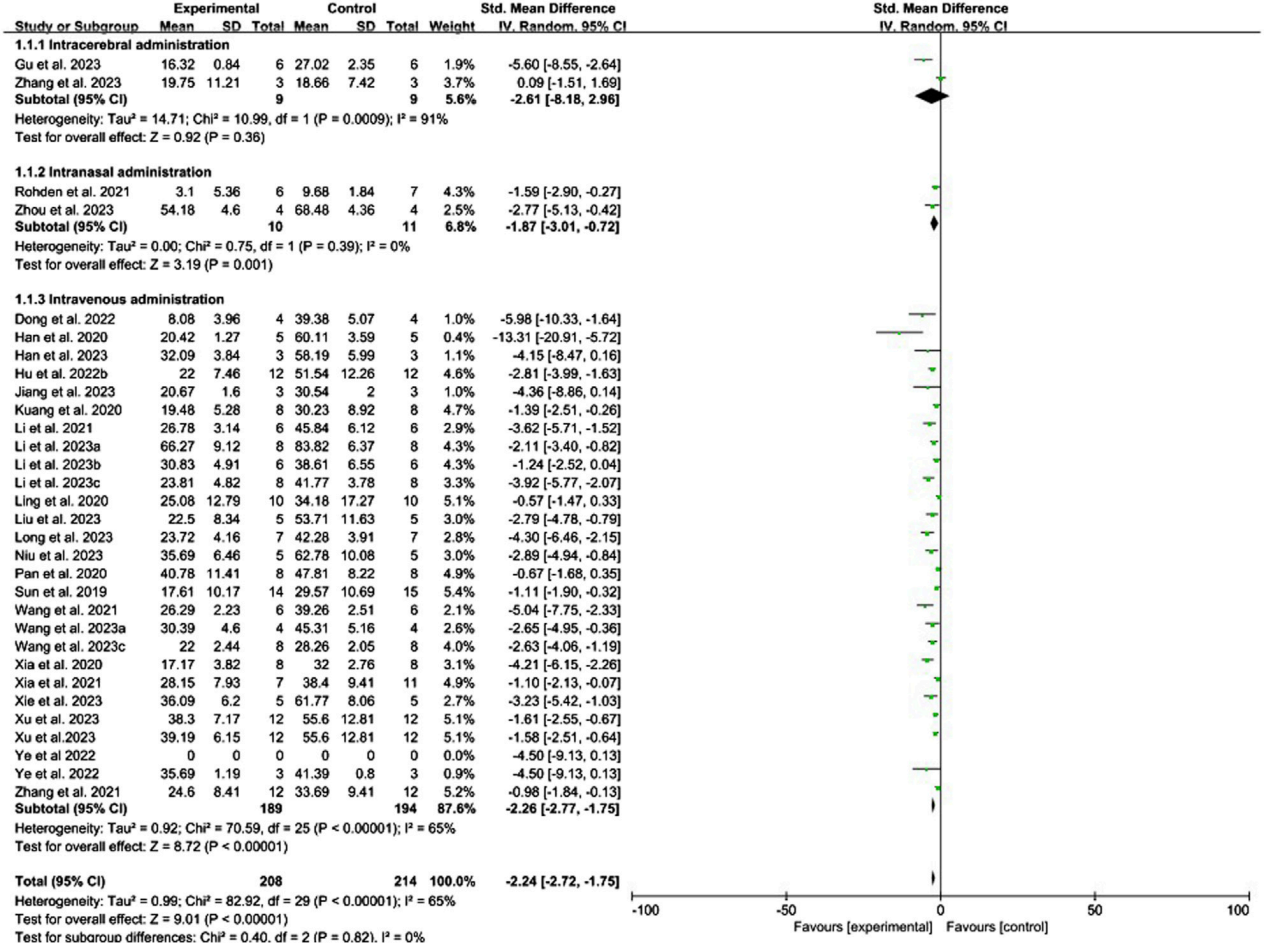
Forest plot for conventional meta-analysis of the effects of routes of administration on the cerebral infarct volume (%).

Conventional meta-analysis of the routes of administration showed (Figure 3) that intranasal (SMD = −1.87, 95% CI [−3.01, −0.72], *p* = 0.001, *I* ^*2*^ = 0%) and intravenous administration (SMD = −2.26, 95% CI [−2.77, −1.75], *p* < 0.001, *I* ^*2*^ = 65%) significantly reduced the cerebral infarct volume (%) compared with that in the negative control group. Intracerebral administration (SMD = −2.61, 95% CI [−8.18, 2.96], *p* = 0.36, *I* ^*2*^ = 91%) did not statistically differ from the negative control group.

**FIGURE 4:**
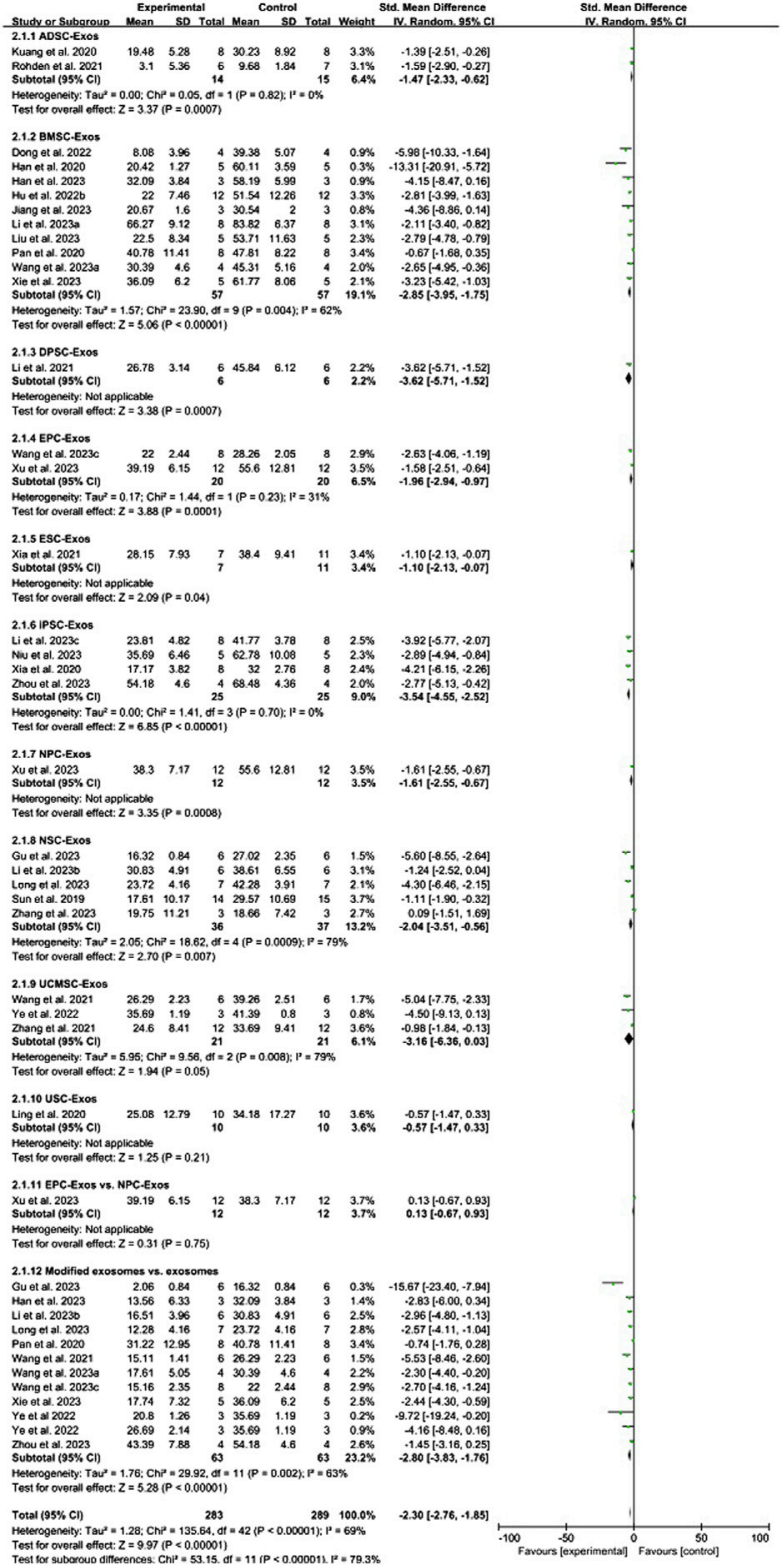
Forest plot for conventional meta-analysis of the effect of stem cell-derived exosomes on cerebral infarct volume (%).

Conventional meta-analysis of stem cell-derived exosomes showed (Figure 4) that ADSC-Exos (SMD = −1.47, 95% CI [−2.33, −0.62], *p* < 0.001, *I* ^*2*^ = 0%), BMSC-Exos (SMD = −2.85, 95% CI [−3.95, −1.75], *p* < 0.001, *I* ^*2*^ = 62%), DPSC-Exos (SMD = −3.62, 95% CI [−5.71, −1.52], *p* < 0.001, *I* ^*2*^ = N/A), EPC-Exos (SMD = −1.96, 95% CI [−2.94, −0.97], *p* < 0.001, *I* ^*2*^ = 31%), ESC-Exos (SMD = −1.10, 95% CI [−2.13, −0.07], *p* < 0.05, *I* ^*2*^ = N/A), iPSC-Exos (SMD = −3.54, 95% CI [−4.55, −2.52], *p* < 0.001, *I* ^*2*^ = 0%), NPC-Exos (SMD = −1.61, 95% CI [-2.55, −0.67], *p* < 0.001, *I* ^*2*^ = N/A), and NSC-Exos (SMD = −2.04, 95% CI [−3.51, −0.56], *p* < 0.01, *I* ^*2*^ = 79%) significantly reduced the cerebral infarct volume (%) compared to the negative control group. UCMSC-Exos (SMD = −3.16, 95% CI [-6.36, 0.03], *p* = 0.05, *I* ^*2*^ = 79%) and USC-Exos (SMD = −0.57, 95% CI [−1.47, 0.33], *p* = 0.21, *I* ^*2*^ = N/A) were not significantly different from those in the negative control group. There was no significant difference between EPC-Exos and NPC-Exos (*p* = 0.75). Compared with unmodified exosomes, modified exosomes (SMD = −2.80, 95% CI [−3.83, −1.76], *p* < 0.001, *I* ^*2*^ = 63%) showed better efficacy.

**FIGURE 5:**
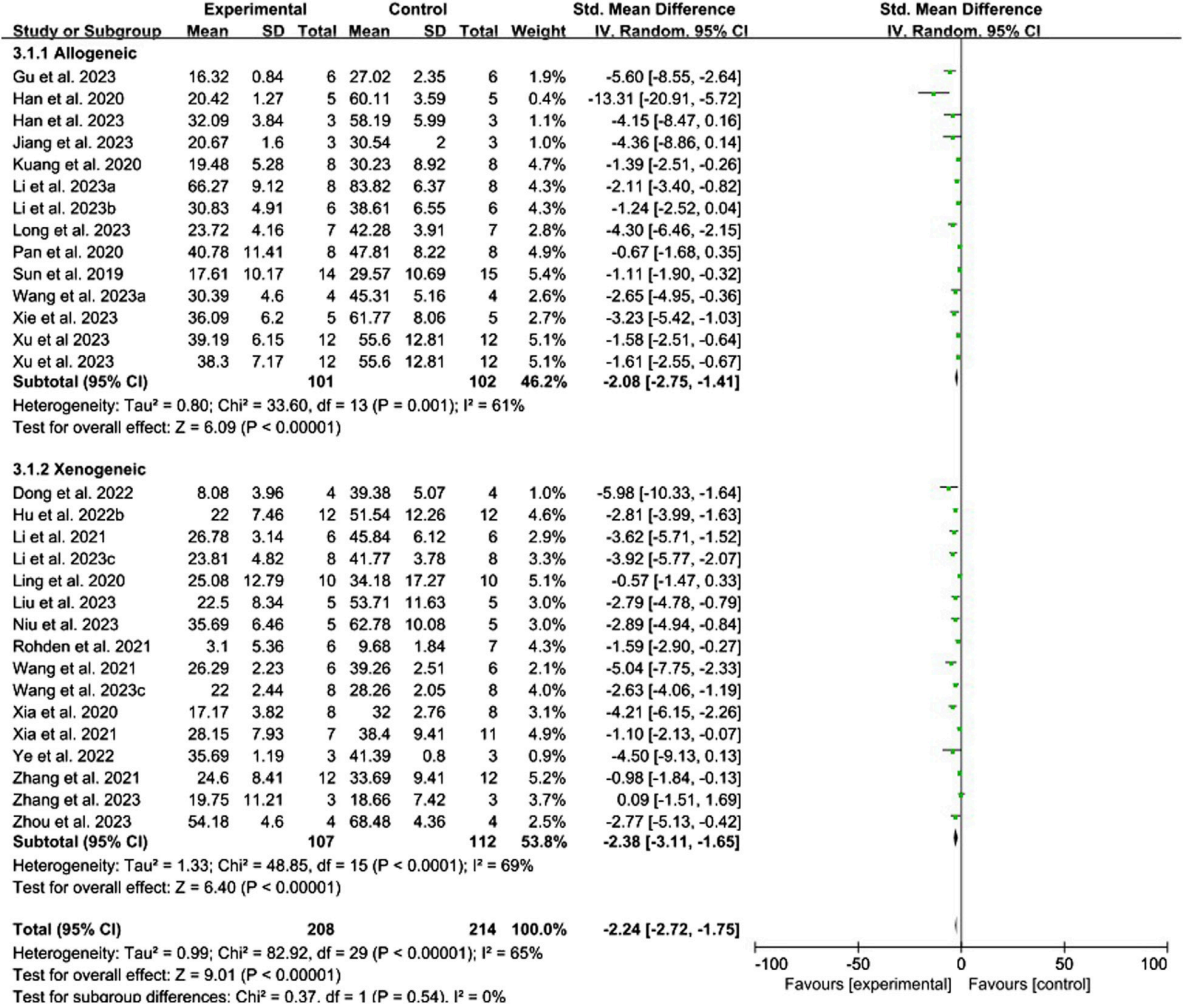
Forest plot for conventional meta-analysis of the effect of immune compatibility on the cerebral infarct volume (%).

Conventional meta-analysis of immune compatibility showed (Figure 5) that both allogeneic (SMD = −2.08, 95% CI [−2.75, −1.41], *p* < 0.001, *I* ^*2*^ = 61%) and xenogeneic (SMD = −2.38, 95% CI [−3.11, −1.65], *p* < 0.001, *I* ^*2*^ = 65%) stem cell-derived exosomes significantly reduced the cerebral infarct volume (%) compared with that in the negative control group.

#### 3.4.2 Network meta-analysis

**FIGURE 6:**
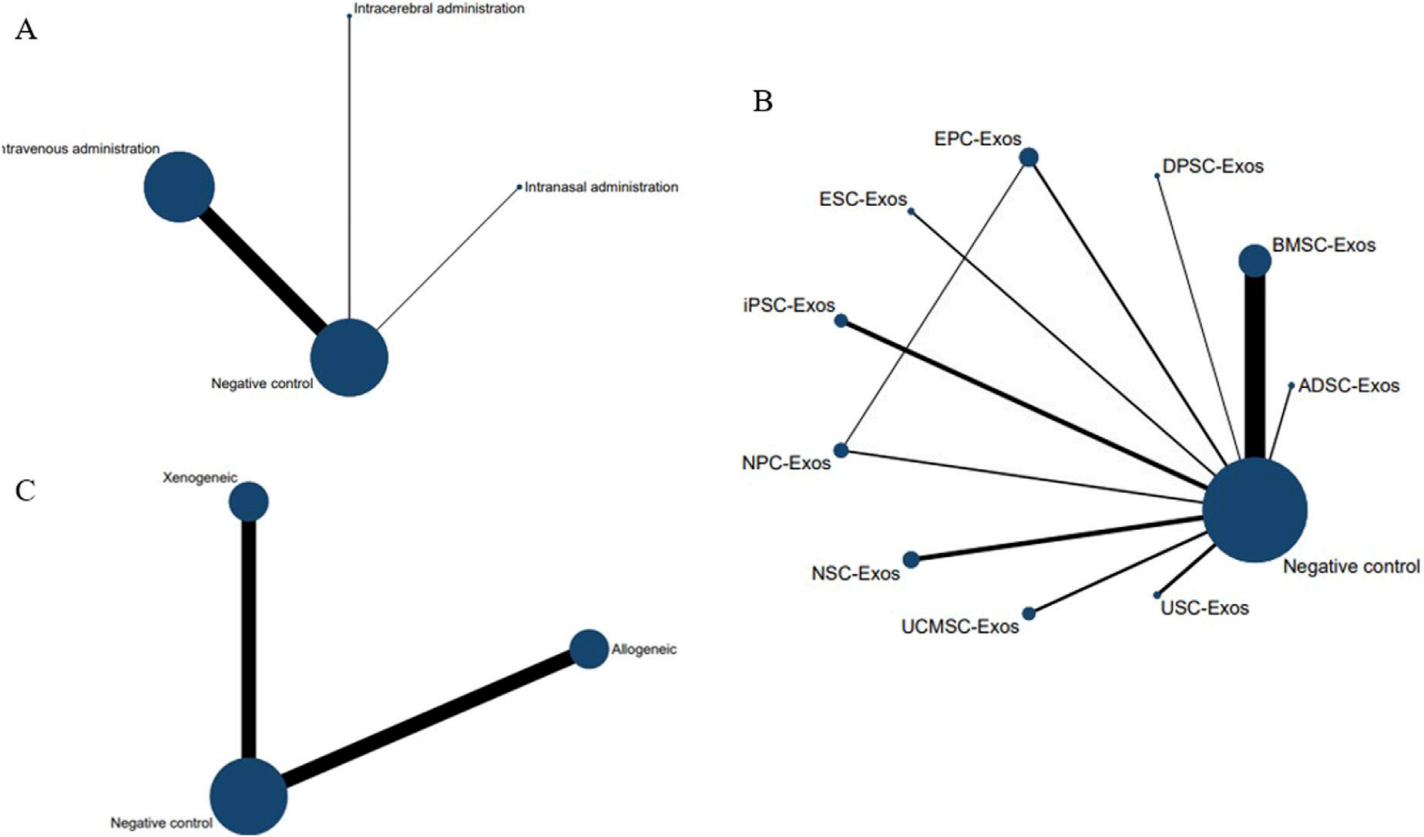
Network evidence maps of the cerebral infarct volume (%). **(A)** Routes of administration; **(B)** Stem cell-derived exosome types; **(C)** Immune compatibility.

**FIGURE 7:**
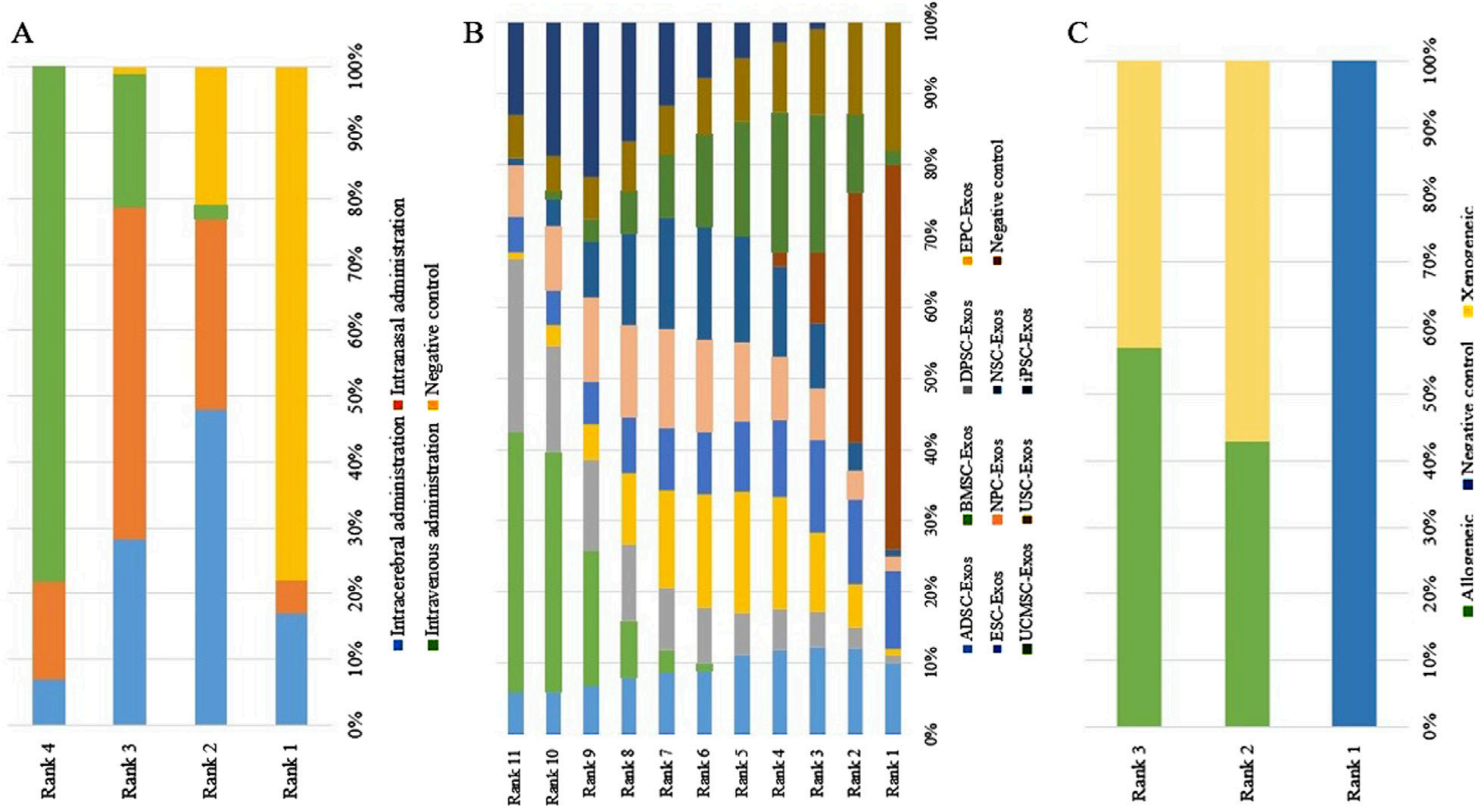
Rank probability ranking plots for the cerebral infarct volume (%). **(A)** Routes of administration; **(B)** Stem cell-derived exosome types; **(C)** Immune compatibility; Rank 1 is the worst, and rank N is the best.

No closed loops appeared in the network evidence plot for the route of administration (Figure 6A), and network meta-analysis was performed via the consistency model. The results showed (Table 1) that there were no significant differences between the various routes of administration. Subgroup analysis of the species showed (Supplementary Table S3) that there were also no significant differences between the various routes of administration in the mouse and rat subgroups. Rank probability ranking showed (Figure 7A; Table 2) that intravenous administration was the best route of administration for reducing the cerebral infarct volume (%), both overall and in the mouse and rat subgroups. Therefore, we next performed a network meta-analysis of the types and immune compatibility of stem cell-derived exosomes after intravenous administration.

**TABLE 1:** Network meta-analysis of routes of administration for reducing the cerebral infarct volume (%).

Intracerebral administration	−3.74 [−23.33, 15.57]	−10.55 [−25.13, 4.21]	6.53 [−7.63, 20.88]
3.74 [−15.57, 23.33]	Intranasal administration	−6.75 [-20.41, 6.58]	10.42 [−3.01, 23.15]
10.55 [−4.21, 25.13]	6.75 [−6.58, 20.41]	Intravenous administration	17.07 [13.29, 20.80]
−6.53 [−20.88, 7.63]	−10.42 [−23.15, 3.01]	−17.07 [−20.80, −13.29]	Negative control

**TABLE 2:** The probability of the best administration route for reducing the cerebral infarct volume (%).

Routes of administration	Total	Mice	Rat
Intracerebral administration	0.07	0.05	-
Intranasal administration	0.15	0.39	0.18
Intravenous administration	0.78	0.56	0.82

Among the 25 studies involving intravenous administration, 10 types of stem cell-derived exosomes were included (ADSC-Exos, BMSC-Exos, DPSC-Exos, EPC-Exos, ESC-Exos, iPSC-Exos, NPC-Exos, NSC-Exos, UCMSC-Exos, and USC-Exos). There was a closed loop in the network evidence map of stem cell-derived exosomes (Figure 6B), and the node splitting method test (Supplementary Table S4) revesled no inconsistency between direct and indirect comparisons (*p* > 0.05); therefore, network meta-analysis was performed via the consistency model. The results (Supplementary Table S5) showed that the efficacy of the BMSC-Exos (SMD = −13.96, 95% CI [−26.10, −1.65]) was superior to that of the UCMSC-Exos, and there was no significant difference between the other types of exosomes. In the mouse and rat subgroups (Supplementary Table S3), there were no significant differences between the various types of stem cell-derived exosomes. Rank probability ranking showed (Figure 7B; Table 3) that BMSC-Exos were the best stem cell-derived exosome for reducing the cerebral infarct volume (%), both overall and in the mouse and rat subgroups.

**TABLE 3:** Probability of the best stem cell-derived exosomes for reducing the cerebral infarct volume (%).

Stem cell-derived exosomes	Total	Mice	Rat
ADSC-Exos	0.06	0.03	—
BMSC-Exos	0.36	0.42	0.36
DPSC-Exos	0.24	0.18	—
EPC-Exos	0.01	0.01	—
ESC-Exos	0.05	0.04	—
NPC-Exos	0.07	0.05	—
NSC-Exos	0.01	0.01	0.28
UCMSC-Exos	0	0.03	0.05
USC-Exos	0.06	—	0.13
iPSC-Exos	0.13	0.24	0.19

No closed loops appeared in the network evidence plot for the immune compatibility of stem cell-derived exosomes (Figure 6C), and network meta-analysis was performed via the consistency model. The results showed (Table 4) that there were no significant differences between allogeneic exosomes and xenogeneic exosomes. In the mouse and rat subgroups (Supplementary Table S3), there was also no significant difference between the two groups. Rank probability ranking showed (Figure 7C; Table 5) that overall, allogeneic exosome efficacy was best; in the mouse subgroup, xenogeneic exosome efficacy was best; and in the rat subgroup, allogeneic exosome efficacy was best.

**TABLE 4:** Network meta-analysis of immune compatibility of stem cell-derived exosomes under tail vein injection for the cerebral infarct volume (%).

Allogeneic	0.60 [-7.19, 8.39]	17.38 [11.79, 22.89]
−0.60 [−8.39, 7.19]	Xenogeneic	16.76 [11.38, 22.22]
−17.38 [−22.89, −11.79]	−16.76 [−22.22, −11.38]	Negative control

**TABLE 5:** Probability of the best immune compatibility for the cerebral infarct volume (%).

Immune compatibility	Total	Mice	Rat
Allogeneic	0.57	0.28	0.76
Xenogeneic	0.43	0.72	0.24

### 3.5 Effect of stem cell-derived exosomes on the mNSS

A total of 21 studies (Li Y. et al., 2023; Jiang et al., 2023; Ye et al., 2022; Dong et al., 2022; Zhao et al., 2020; Zhang et al., 2020; Xia et al., 2020; Mahdavipour et al., 2020; Ling et al., 2020; Li et al., 2020; Moon et al., 2019; Liang et al., 2024; Zhou et al., 2023; Zhang et al., 2023; Xie et al., 2023; Wang et al., 2023b; Li R. et al., 2023; Gu et al., 2023; Campero-Romero et al., 2023; Hu X. et al., 2022; Li et al., 2021) reported the mNSS, which included 214 rats and 173 mice, and included 8 types of exosomes (ADSC-Exos, BMSC-Exos, DPSC-Exos, iPSC-Exos, NPC-Exos, NSC-Exos, UCMSC-Exos, and USC-Exos). Thirteen studies used intravenous administration (tail vein injection), 2 studies used intranasal administration, and 6 studies used intracerebral administration (5 lateral ventricular injections and 1 localized striatal graft). Thirteen studies used allogeneic exosomes and 8 studies used xenogeneic exosomes. A total of 9 studies used engineered modified exosomes, with 2 studies endogenously loading non-coding RNAs, 2 studies endogenously loading RNAs, 3 studies pretreating stem cells (including brain tissue extracts, interferon-gamma, LBP), and 2 studies surface-modifying exosomes (RGD/Angiopep-2 peptide, hyaluronic acid hydrogel).

#### 3.5.1 Conventional meta-analysis

**FIGURE 8:**
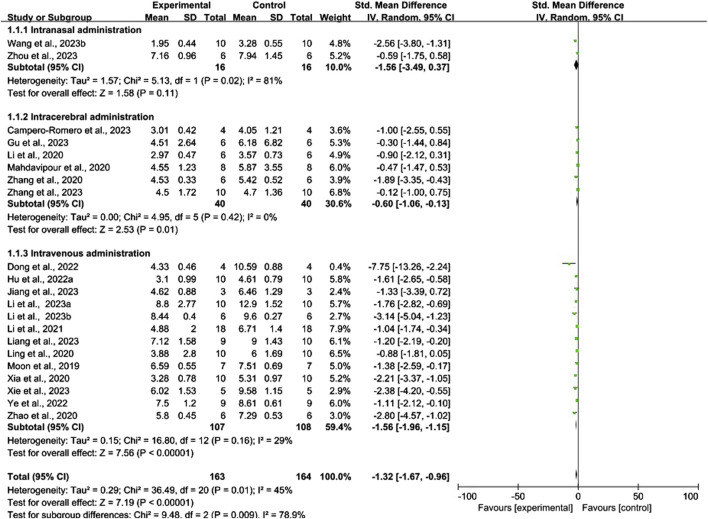
Forest plot for conventional meta-analysis of the effects of routes of administration on the mNSS.

Conventional meta-analysis of routes of administration showed (Figure 8) that intravenous (SMD = −1.56, 95% CI [−1.96, −1.15], *p* < 0.001, *I* ^*2*^ = 29%) and intracerebral administration (SMD = −0.60, 95% CI [−1.06, −0.13], *p* < 0.05, *I* ^*2*^ = 0%) significantly reduced the mNSS compared with that of negative controls. The effects of intranasal administration (SMD = −1.56, 95% CI [−3.49, 0.37], *p* = 0.11, *I* ^*2*^ = 81%) were not significantly different from those of the negative controls.

**FIGURE 9:**
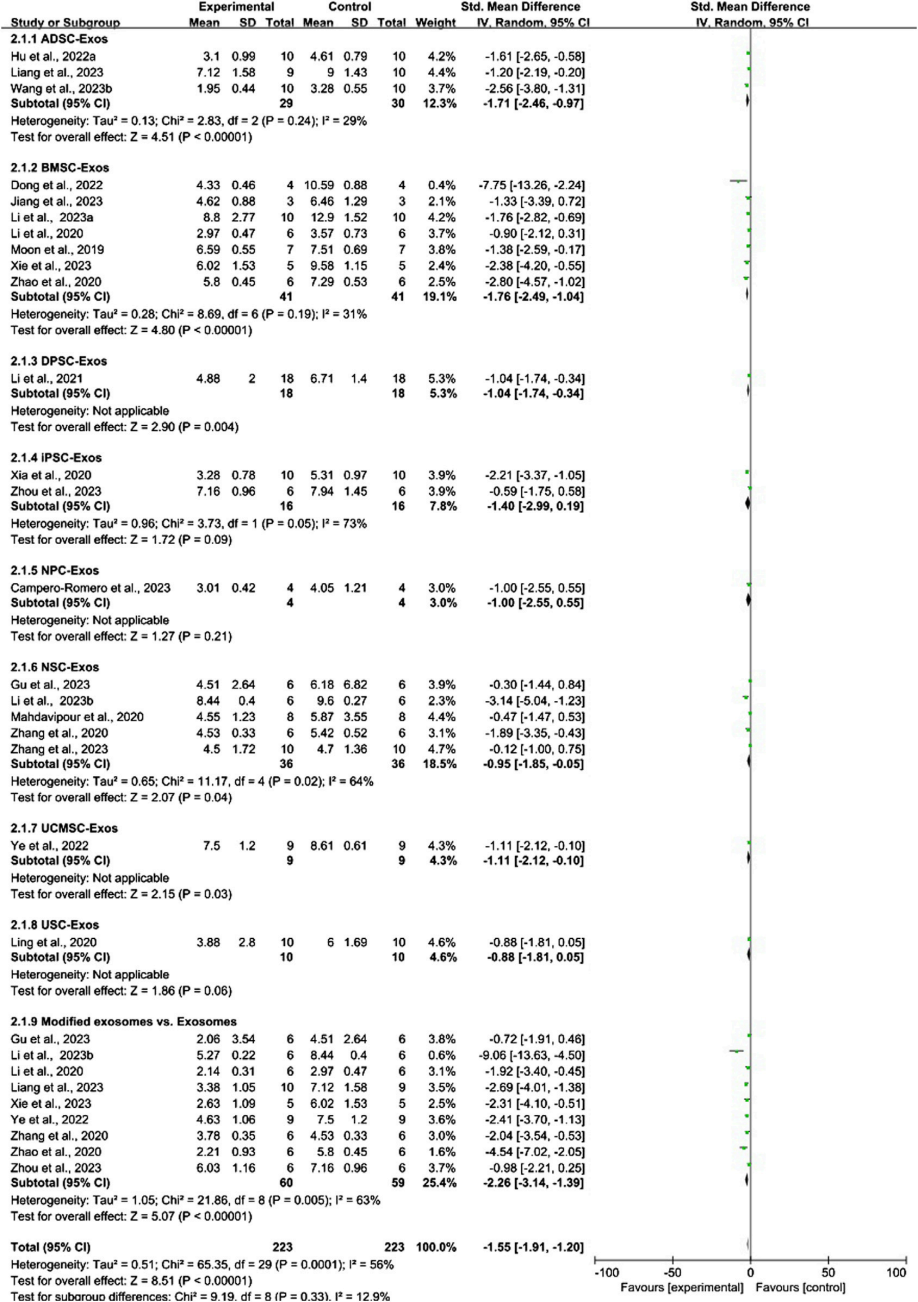
Forest plot for conventional meta-analysis of the effects of stem cell-derived exosomes on the mNSS.

Conventional meta-analysis of stem cell-derived exosomes showed (Figure 9) that ADSC-Exos (SMD = −1.71, 95% CI [−2.46, −0.97], *p* < 0.001, *I* ^*2*^ = 29%), BMSC-Exos (SMD = −1.76, 95% CI [-2.49, −1.04], *p* < 0.001, *I* ^*2*^ = 31%), DPSC-Exos (SMD = −1.04, 95% CI [−1.74, −0.34], *p* < 0.05, *I* ^*2*^ = N/A), NSC-Exos (SMD = −0.95, 95% CI [−1.85, −0.05], *p* < 0.05, *I* ^*2*^ = 64%), and UCMSC-Exos (SMD = −1.11, 95% CI [−2.12, −0.10], *p* < 0.05, *I* ^*2*^ = N/A) significantly reduced the mNSS compared with that of the negative controls. iPSC-Exos (SMD = −1.40, 95% CI [−2.99, 0.19], *p* = 0.09, *I* ^*2*^ = 73%), NPC-Exos (SMD = −1.00, 95% CI [−2.55, 0.55], *p* = 0.21, *I* ^*2*^ = N/A), and USC-Exos (SMD = −0.88, 95% CI [−1.81, 0.05], *p* = 0.06, *I* ^*2*^ = N/A) were not significantly different from the negative controls. Compared with unmodified exosomes, modified exosomes (SMD = −2.26, 95% CI [−3.14, −1.39], *p* < 0.001, *I* ^*2*^ = 63%) had better efficacy.

**FIGURE 10:**
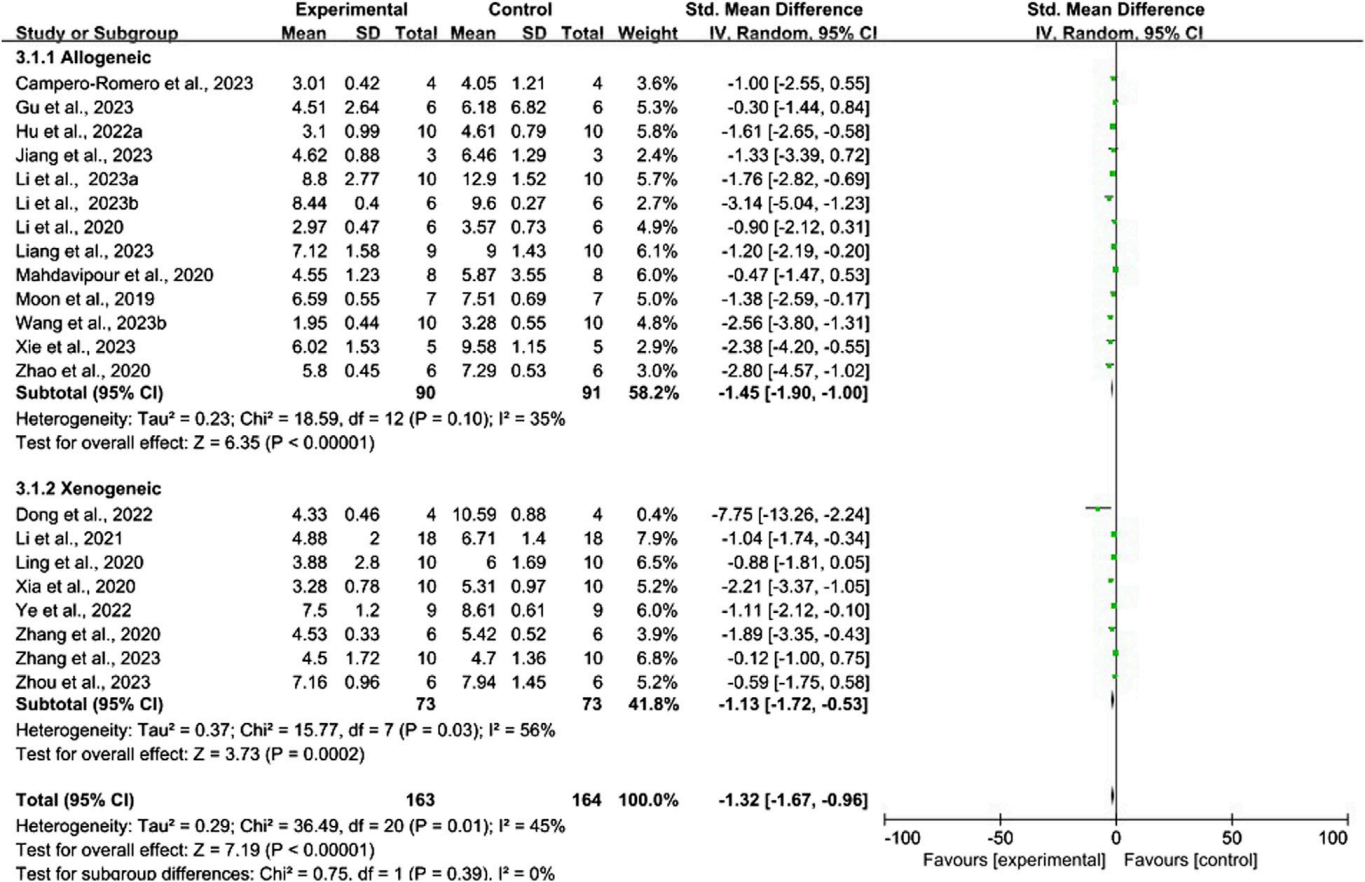
Forest plot for conventional meta-analysis of immune compatibility on the mNSS.

Conventional meta-analysis of the immune compatibility of stem cell-derived exosomes showed (Figure 10) that both allogeneic exosomes (SMD = −1.45, 95% CI [−1.90, −1.00], *p* < 0.001, *I* ^*2*^ = 35%) and xenogeneic exosomes (SMD = −1.13, 95% CI [−1.72, −0.53], *p* < 0.001, *I* ^*2*^ = 56%) significantly reduced the mNSS compared with that of negative controls.

#### 3.5.2 Network meta-analysis

**FIGURE 11:**
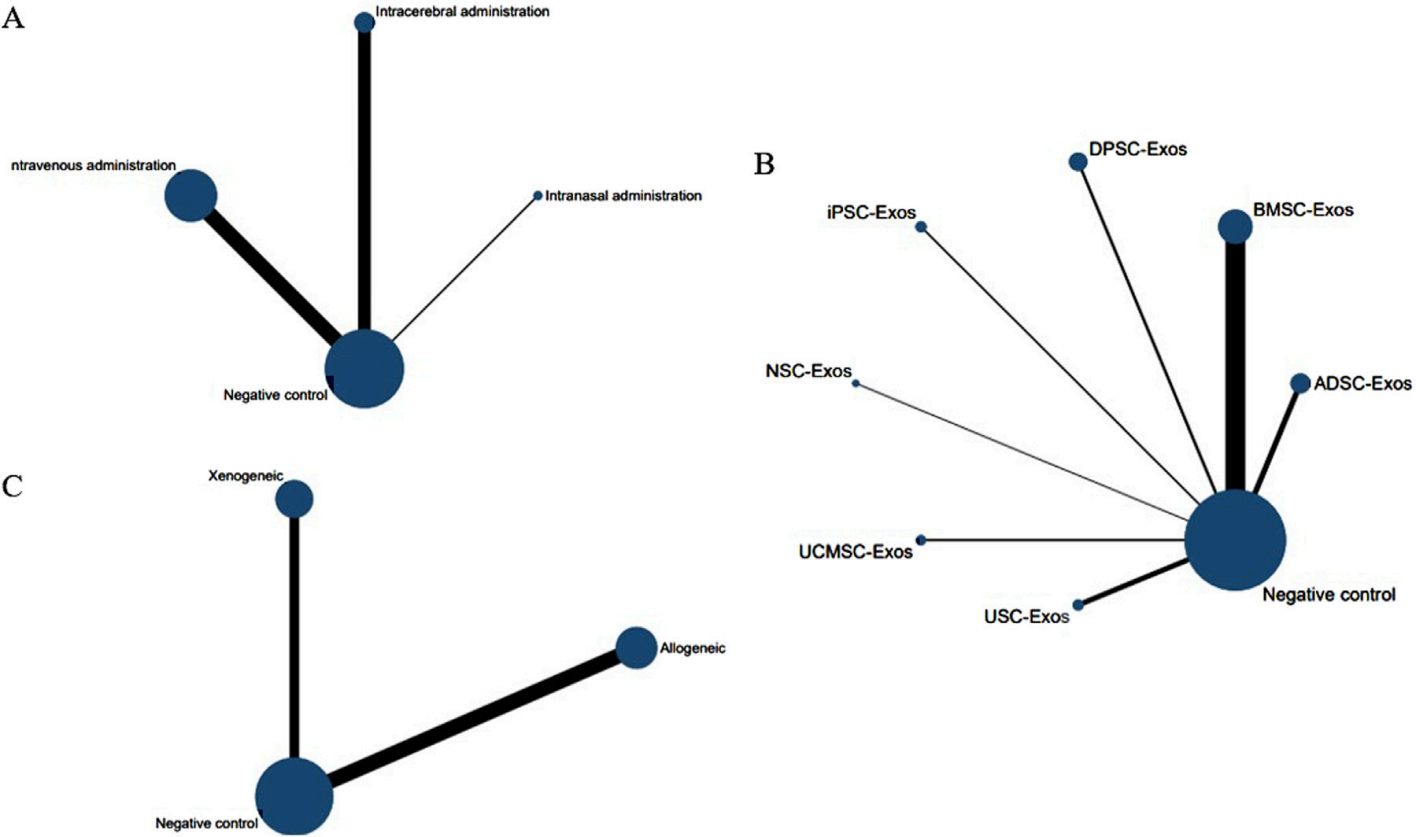
Network evidence maps for the mNSS. **(A)** Routes of administration; **(B)** Stem cell-derived exosome types; **(C)** Immune compatibility.

**FIGURE 12:**
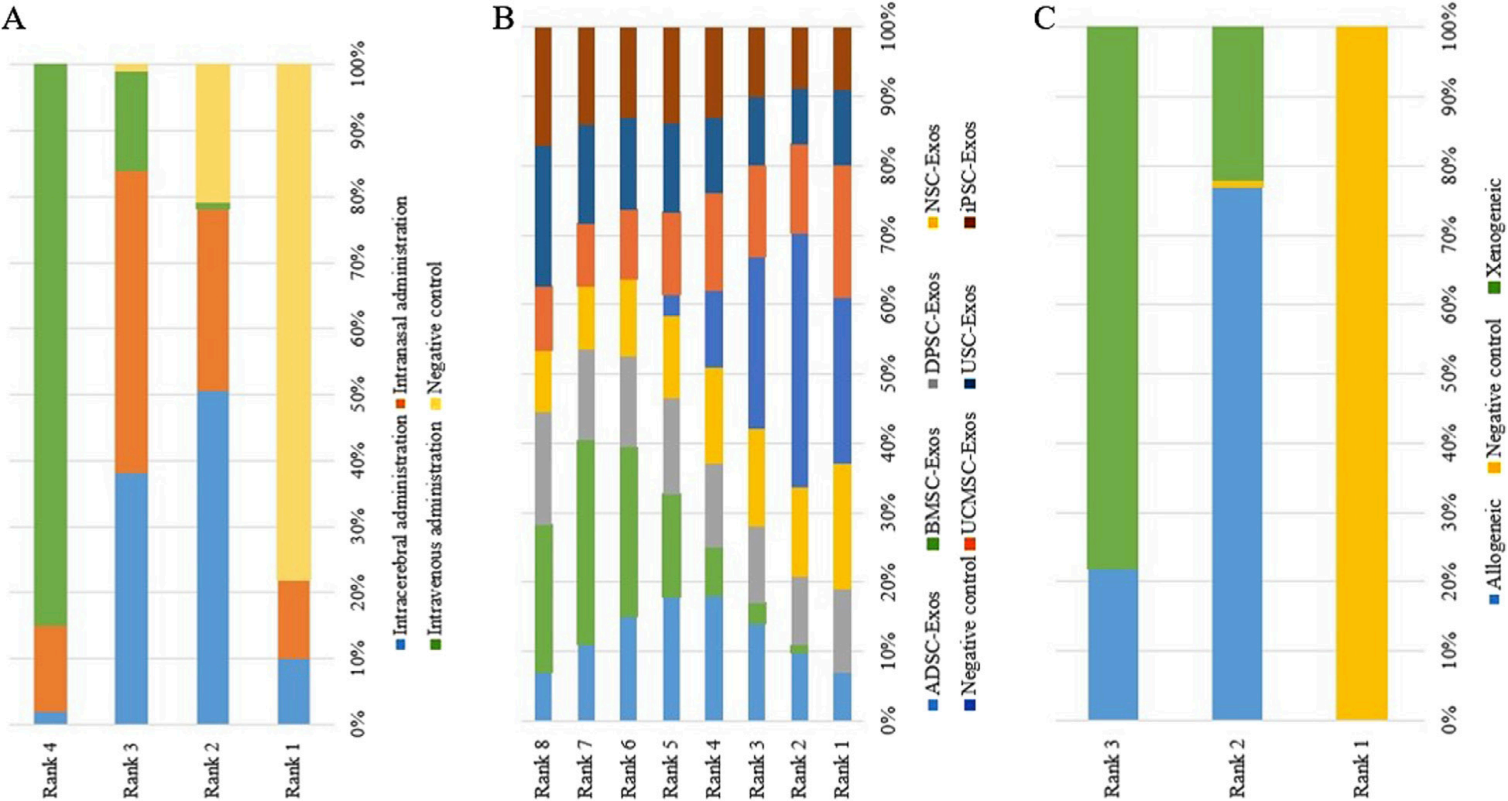
Rank probability ranking plots for the mNSS. **(A)** Routes of administration; **(B)** Stem cell-derived exosome types; **(C)** Immune compatibility; Rank 1 is the worst, and rank N is the best.

No closed loops appeared in the network evidence plot for routes of administration (Figure 11A), and network meta-analysis was performed via the consistency model. The results showed (Table 6) that there were no significant differences between the various routes of administration. In the mouse and rat subgroups (Supplementary Table S6), there were also no significant differences between the various routes of administration. Rank probability ranking showed (Figure 12A; Table 7) that intravenous administration was the best route of administration for reducing the mNSS, both overall and in the mouse and rat subgroups. Therefore, we next performed a network meta-analysis of the types and immune compatibility of stem cell-derived exosomes after intravenous administration.

**TABLE 6:** Network meta-analysis of routes of administration for reducing the mNSS.

Intracerebral administration	−0.28 [−2.68, 2.03]	−1.43 [−2.92, 0.02]	0.80 [−0.48, 2.03]
0.28 [−2.03, 2.68]	Intranasal administration	−1.12 [-3.27, 0.99]	1.08 [−0.87, 3.05]
1.43 [−0.02, 2.92]	1.12 [−0.99, 3.27]	Intravenous administration	2.21 [1.43, 3.01]
−0.80 [−2.03, 0.48]	−1.08 [−3.05, 0.87]	−2.21 [−3.01, −1.43]	Negative control

**TABLE 7:** The probability of the best administration route for mNSS.

Routes of administration	Total	Mice	Rat
Intracerebral administration	0.02	0.07	0.12
Intranasal administration	0.13	0.20	-
Intravenous administration	0.85	0.73	0.88

Seven types of stem cell-derived exosomes (ADSC-Exos, BMSC-Exos, DPSC-Exos, iPSC-Exos, NSC-Exos, UCMSC-Exos, and USC-Exos) were included in the 13 studies involving intravenous administration. There were no closed loops in the network evidence map (Figure 11B), and network meta-analysis was performed via the consistency model. The results (Supplementary Table S7) showed that there were no significant differences between the various types of stem cell-derived exosomes. There were also no significant differences between the various types of stem cell-derived exosomes in the mouse and rat subgroups (Supplementary Table S6). Rank probability ranking showed (Figure 12B; Table 8) that BMSC-Exos were the best stem cell-derived exosomes for reducing the mNSS, both overall and in the mouse and rat subgroups.

**TABLE 8:** Probability of the best stem cell-derived exosomes for mNSS.

Stem cell-derived exosomes	Total	Mice	Rat
ADSC-Exos	0.07	0.07	-
BMSC-Exos	0.21	0.72	0.31
DPSC-Exos	0.16	0.14	-
NSC-Exos	0.09	0.06	-
UCMSC-Exos	0.09	-	0.15
USC-Exos	0.20	-	0.28
iPSC-Exos	0.17	-	0.25

No closed loops were present in the network evidence plot for immune compatibility (Figure 11C), and network meta-analysis was performed via the consistency model. The results showed (Table 9) that there were no significant differences between allogeneic exosomes and xenogeneic exosomes. In the mouse and rat subgroups (Supplementary Table S6), there was also no significant difference between the two groups. Rank probability ranking showed (Figure 12C; Table 10) that xenogeneic stem cell-derived exosomes had the best efficacy in reducing the mNSS, both overall and in the mouse and rat subgroups.

**TABLE 9:** Network meta-analysis of immune compatibility of stem cell-derived exosomes under tail vein injection for the mNSS.

Allogeneic	−0.76 [−2.76, 1.33]	1.93 [0.69, 3.28]
0.76 [−1.33, 2.76]	Xenogeneic	2.70 [1.05, 4.30]
−1.93 [−3.28, −0.69]	−2.70 [−4.30, −1.05]	Negative control

**TABLE 10:** Probability of the best immune compatibility for the mNSS.

Immune compatibility	Total	Mice	Rat
Allogeneic	0.22	0.47	0.28
Xenogeneic	0.78	0.52	0.72

### 3.6 Publication bias

**FIGURE 13:**
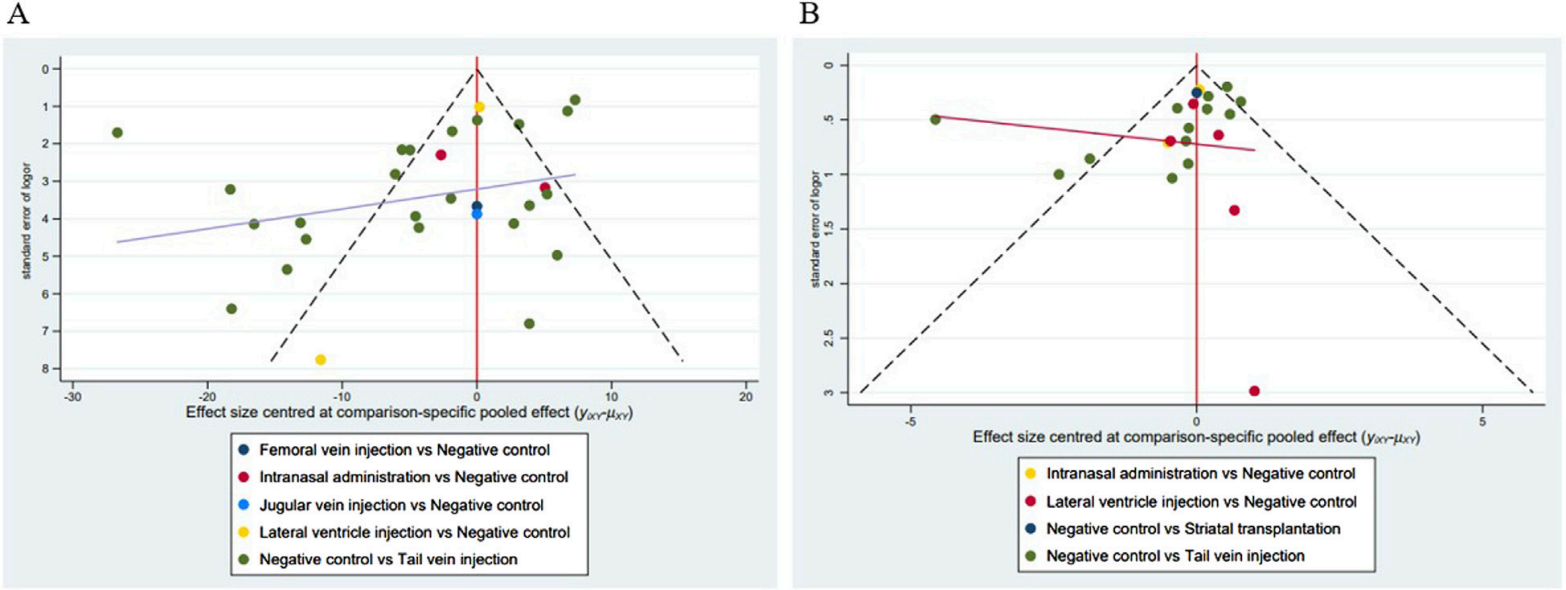
Funnel plots of the included studies. **(A)** Studies reporting the cerebral infarct volume (%); **(B)** Studies reporting the mNSS.

Funnel plots, Begg’s test (*p* < 0.05), and Egger’s test (*p* < 0.05) results showed (Figure 13; Table 11) that publication bias and small-sample effects may be present in studies reporting the cerebral infarction volume (%) and the mNSS.

**TABLE 11:** Tests for publication bias.

Outcomes	Number of studies	Begg’s test		Egger’s test	
		*Z*	*P*	*T*	*P*
Infarct volume%	29	4.37	0.000	−8.33	0.000
mNSS	21	3.23	0.001	−4.09	0.001

## 4 Discussion

A total of 38 randomized controlled animal experiments were included in this study, in which the cerebral infarct volume (%) and the mNSS were used as outcome indicators. First, we clarified the efficacy of various stem cell-derived exosomes for various routes of administration, types, and immune compatibility in a rat/mouse ischemic stroke model via conventional meta-analysis. Compared with the negative control group, 1) intravenous administration significantly reduced the cerebral infarct volume (%) and mNSS; intranasal administration significantly reduced the cerebral infarct volume (%); and intracerebral administration significantly reduced the mNSS. 2) ADSC-Exos, BMSC-Exos, DPSC-Exos, and NSC-Exos significantly reduced the cerebral infarct volume (%) and mNSS; EPC-Exos, ESC-Exos, iPSC-Exos and NPC-Exos significantly reduced the cerebral infarct volume (%); UCMSC-Exos significantly reduced the mNSS; there was no significant difference between USC-Exos and negative controls; and modified exosomes had better efficacy than unmodified exosomes. 3) Both allogeneic and xenogeneic stem cell-derived exosomes significantly reduced the cerebral infarct volume (%) and the mNSS.

We subsequently compared the efficacy of various routes of administration, types, and immune compatibility of stem cell-derived exosomes via network meta-analysis and screened the optimal delivery strategies by rank probability ranking. 1) Compared with the various routes of administration, intravenous administration was the best route of administration for reducing the cerebral infarct volume (%) and the mNSS, both overall and in the mouse and rat subgroups. 2) Among the 10 types of stem cell-derived exosomes that were intravenously administered, BMSC-Exos were the best stem cell-derived exosomes for reducing the cerebral infarct volume (%) and mNSS, both overall and in the rat and mouse subgroups. 3) Overall and in rat subgroups, allogeneic exosomes had the best efficacy in reducing the cerebral infarct volume (%), whereas in the mouse subgroup, xenogeneic exosomes had the best efficacy. Both overall and in the mouse and rat subgroups, xenogeneic stem cell-derived exosomes had the best efficacy in reducing the mNSS.

The route of administration is closely related to the stage of ischemic stroke, drug biodistribution, efficacy, and safety, and is one of the most controversial issues in preclinical and clinical studies. Our study clarified the efficacy of stem cell-derived exosomes administered intravenously, intranasally, and intracerebrally, and revealed that intravenous administration was the optimal route of administration. Compared with intracerebral administration, intravenous administration is considered to be the most direct and least invasive technique; it is more suitable for the early treatment of ischemic stroke and is the most frequently used route of administration in the clinic (Taussky et al., 2011; Rascón-Ramírez et al., 2021). Two recent meta-analyses confirmed that intravenous administration is the safest route of administration for patients with ischemic stroke and that intracerebral administration increases the incidence of adverse events (Fauzi et al., 2023; Wang et al., 2020). Studies have shown that stem cell-derived exosomes accumulate in large quantities in organs such as the spleen, lungs, liver, and kidneys after intravenous administration, with less distribution in the brain (Li Y. et al., 2021; Song et al., 2023). Toxicological studies have shown that the accumulation of stem cell-derived exosomes does not cause toxic damage to these organs or significant changes in biochemical blood parameters (Rodrigues et al., 2021). Intranasal administration allows for the direct delivery of drugs to the brain via the olfactory region, thus increasing the bioavailability and transport efficiency of the drug (Gotoh et al., 2024). Betzer et al. (Betzer et al., 2017) found that 1 h after administration in cerebral ischemic mice, exosomes administered intranasally were more than twice as abundant in the brain as those administered intravenously. Our results showed that intranasal administration of stem cell-derived exosomes significantly reduced the cerebral infarct volume (%), but the effect of reducing the mNSS was not significantly different from that of the negative control group. This evidence suggests that intranasal administration is a promising noninvasive route for treating ischemic stroke, but more experimental data are still needed to confirm the efficacy and safety of intranasal administration.

This study showed that among the 10 types of exosomes, the BMSC-Exos had the best efficacy after intravenous administration. BMSC-Exos are currently the most widely used exosomes for treating central nervous system (CNS) diseases. The advantages of BMSCs over other stem cells and the progress of the use of BMSC-Exos in CNS diseases such as ischemic stroke have been well described in several recently published systematic reviews (Chai et al., 2024; Cai et al., 2020; Chu et al., 2020; Pratiwi et al., 2024). In addition, we found that engineered modified exosomes were more efficacious than unmodified exosomes. Exosomes mainly play a key role in regulating biological functions by delivering proteins, non-coding RNAs, and other contents (Pan et al., 2023). Nine of the included studies transfected stem cell-derived exosomes with non-coding RNAs with cerebral protective effects, which enhanced the effects of stem cell-derived exosomes on the inhibition of neuroinflammation, anti-apoptosis, anti-oxidative stress, and neovascularization. Moreover, the efficacy of exosomes can be enhanced by modulating the microenvironment of their source cell culture (Haupt et al., 2023). In this meta-analysis, five studies pretreated stem cells with cerebral infarction tissue extracts, proinflammatory factors, drug-containing serum, and small-molecule drugs, and the resulting exosomes had a greater cerebral protective effect. However, the exact molecular mechanism by which preconditioned exosomes ameliorate cerebral infarction remains unclear. Although stem cell-derived exosomes are promising for the treatment of ischemic stroke, the lack of brain-targeting ability after systemic administration has been one of the bottlenecks limiting their efficacy. The study notes that exosomes can be engineered with modifications that alter their *in vivo* biodistribution, including enhanced brain targeting (Choi et al., 2021). Studies continue to confirm that surface-engineered modification of exosomes can significantly increase brain content and improve bioavailability (Tian et al., 2018; Tian et al., 2021). In this meta-analysis, 2 studies used surface-engineered modifications of exosomes. Liang et al. (Liang et al., 2024) utilized the brain-targeting ability of the RGD peptide and the cell-penetrating ability of the Angiopep-2 peptide for dual surface modification of MSC-derived exosomes. After intravenous administration, the modified exosomes rapidly accumulated in the ischemic region of the brain and targeted ischemic vessels. Gu et al. (2023) used adhesive hyaluronic acid hydrogels loaded with exosomes to achieve continuous delivery and slow release of exosomes in the ischemic region of the brain, increasing the duration of action of exosomes in the brain, which in turn enhanced their efficacy in ameliorating cerebral infarction.

The low immunogenicity of exosomes opens up the possibility of heterologous applications, but it is not clear whether there are differences in therapeutic efficacy across species. An experimental study by Dong et al. (2020) showed that both allogeneic exosomes and xenogeneic exosomes promoted soft tissue repair, but there was no significant difference between the two methods. Our study also demonstrated no significant difference between allogeneic stem cell-derived exosomes and xenogeneic exosomes in reducing the cerebral infarct volume (%) and the mNSS. However, the rank probability showed that allogeneic stem cell-derived exosomes had the best efficacy in reducing the cerebral infarct volume (%), whereas xenogeneic stem cell-derived exosomes had the best efficacy in reducing the mNSS. Many factors contribute to this controversy. Different species and different cellular sources lead to differences in exosome contents that can perform immunostimulatory or immunosuppressive functions (Fujii et al., 2018). In addition, even exosomes of the same cellular origin can elicit different immunomodulatory effects depending on their culture environment and formulation (Kordelas et al., 2019; Lia et al., 2020). In addition to efficacy, safety considerations that may arise from the intra- and inter-species application of exosomes must be taken into account, as this is critical for the clinical translation of exosomes. Unfortunately, none of the 38 included experimental animal studies reported the occurrence of adverse events. Dehghani et al. (2022) conducted a randomized controlled clinical trial showing that patients with malignant middle cerebral artery occlusion did not develop local hematomas or adverse effects after injection of allogeneic stem cell-derived exosomes, suggesting that allogeneic exosome therapy is safe. A recent meta-analysis of clinical trials on exosomes showed no significant difference in the incidence of adverse events between allogeneic *versus* autologous administration, nor between engineered modified *versus* unmodified exosomes, and that, in general, exosome-based therapy is safe and feasible for patients (Van Delen et al., 2024). Our meta-analysis indirectly compares the efficacy of allogeneic stem cell-derived exosomes with xenogeneic exosomes by integrating existing evidence, which could provide options for preclinical studies, but more direct comparative evidence is still needed to validate the results. Future studies should also emphasize the safety of the exosome administration.

Notably, the dose and duration of exosome administration (Supplementary Table S2) were also important factors influencing the cerebral infarct volume (%) and mNSS. However, these factors were too heterogeneous across studies for quantitative meta-analysis, and we performed a qualitative review of them. 1) In terms of dose administered, among the included studies, some used weight units (μg), and some used particle counts (particles); there is no consensus on the dose to be administered for exosomes, and comparing doses from different studies is challenging. Our study found a range of 10–300 μg (or 2 × 10^6^–3 × 10^11^ particles) for intravenous administration, 800 ng - 100 μg (or 4 × 10^9^–1 × 10^10^ particles) for intracerebral administration, and 10 μg–200 μg/kg for intranasal administration. These suggest that the effective dose of stem cell-derived exosomes range is wide. Both Moon et al. (2019) and Hu et al. (Hu H. et al., 2022) showed that intravenous injection of BMSC-Exos promoted neurovascular neovascularization and neurological function recovery in cerebral ischemic rats exhibited dose-dependent effects (10, 25, 30, 50, 100, and 300 μg/rat), with an optimal dose of 100 μg/rat for promotion of neovascularization. Rohden et al. (2021) showed that the transnasal administration of ADSC-Exos to promote neurological recovery also showed a dose-dependent effect (100, 200, and 300 μg/kg), and an administration dose of 200 μg/kg resulted in complete neurological recovery in MCAO/R rats. However, the doses in these studies are within the range of safe doses, and toxicological studies are lacking, so more preclinical studies are needed to optimize the dose of exosomes administered. 2) In terms of courses of treatment, of the included studies, 24 were single-dose administrations with a maximum observation period of up to 2 months; 9 were once-daily administrations with a range of 2–28 days; 2 were 2-weekly administrations with a course of 28 days; and 1 was twice-daily administrations for 3 consecutive days, but with an observation period of up to 28 days 1 was 3 times-daily administrations for 7 consecutive days. As can be seen, the current preclinical studies on the frequency and courses of administration of stem cell-derived exosomes are very variable, with observation times covering the acute, subacute, and recovery phases of ischemic stroke. Among them, BMSC-Exos significantly reduced the cerebral infarct volume (%) and mNSS on days 1, 14, and 28 after a single administration via intravenous injection. We believe that in the future studies should focus on the short-term efficacy and long-term prognosis of exosomes in the treatment of ischemic stroke and enhance the standardization and normalization of this therapy.

This meta-analysis has several limitations: 1) Due to the different units (particles, μg, μl) of exosomes administered in the included studies, we did not further screen for the optimal dose to be administered. 2) We did not compare proteins, genes, and cytokines at the molecular level because different studies differ in their methods of detection or in the tissues from which they were taken. (3) Because of species differences and differences in follow-up time between studies, as well as funnel plots, Begg’s test, and Egger’s test indicating possible publication bias and small-sample effects, these factors call for caution in interpreting the results of meta-analyses.

## 5 Conclusion

We found that various routes of administration, types, and immune compatibility of stem cell-derived exosomes were efficacious in ischemic stroke animal models via conventional and network meta-analyses of 38 randomized controlled animal experiments. Among them, intravenous administration is the best route of administration, BMSC-Exos are the best stem cell-derived exosome type, allogeneic exosomes have the best efficacy in reducing the cerebral infarct volume (%), and xenogeneic exosomes have the best efficacy in reducing the mNSS, which can provide options for preclinical studies. Considering the limitations of this meta-analysis and the risk of uncertainty in the experimental design, outcome measurement, and reporting of the current studies, more high-quality randomized controlled animal experiments, especially direct comparative evidence, are needed in the future to determine the optimal administration strategy of stem cell-derived exosomes for treating ischemic stroke.

## Data Availability

The original contributions presented in the study are included in the article/Supplementary Material, further inquiries can be directed to the corresponding author.
