# Supplementary material for: Stem cell-derived exosomes for ischemic stroke: a conventional and network meta-analysis based on animal models
Source: Front Pharmacol. 2024 Oct 23;15:1481617. doi: 10.3389/fphar.2024.1481617 (PMC11537945; doi:10.3389/fphar.2024.1481617)
Supplement: Supplementary file 1 [file Table1.docx]

| Supplementary Table S1 Characteristics of the included studies | **Outcome** |  |  | 1. ② | ①② |
| --- | --- | --- | --- | --- | --- |
|  | **Dose** | 200 μg | 100 μg | 1×10^10^ particles | 100 μg |
|  | **Way of administrated** | Tail vein injection | Tail vein injection | Tail vein injection | Tail vein injection |
|  | **Exosome Diameter** | 50~150 nm | 50~150 nm | 145 nm | - |
|  | **Type of exosomes** | SD rat BMSC-Exos | SD rat NSC-Exos | SD rat BMSC-Exos | SD rat BMSC-Exos |
|  | **Group(n)** | MCAO/R+PBS;  MCAO/R+Exos;  MCAO/R+miR-193b-5p Exos;  MCAO/R+miR inhibitor Exos. | MCAO/R+PBS;  MCAO/R+Exos;  MCAO/R+BHD Exos. | pMCAO+PBS;  pMCAO+Exos | MCAO/R+vehicle(17);  MCAO/R+Exos(17). |
|  | **Age** | - | 2.5~3 months | 8 weeks | 2 months |
|  | **Sex** | male | male | male | male |
|  | **Species** | SD rat | SD rat | SD rat | SD rat |
|  | **Location** | China | China | China | China |
|  | **Reference** | Wang et al., 2023a | Long et al., 2023 | Li et al., 2023a | Jiang et al., 2023 |

| Supplementary Table S1 (continued) | **Outcome** | ①② | 1. ② |  |  |
| --- | --- | --- | --- | --- | --- |
|  | **Dose** | 80 μg | 3×10^11^ particles | 100 μg | 200 μg/Kg |
|  | **Way of administrated** | Tail vein injection | Tail vein injection | Tail vein injection | Intranasal administration |
|  | **Exosome Diameter** | 50~120 nm | 100 nm | 30~100 nm | 140 nm |
|  | **Type of exosomes** | human UCMSC-Exos | human BMSC-Exos | human UCMSC-Exos | human ADSC-Exos |
|  | **Group(n)** | MCAO/R+PBS(35);  MCAO/R+Exos(35);  MCAO/R+N Exos(35);  MCAO/R+I Exos(35). | pMCAO+PBS(4);  pMCAO+Exos(4);  pMCAO+miR-23a-3p inhibitor Exos(4). | MCAO/R+PBS(17);  MCAO/R+Exos(17);  MCAO/R+miR-24 Exos(17);  MCAO/R+miR inhibitor Exos(17). | pMCAO+PBS;  pMCAO+Exos. |
|  | **Age** | 8 weeks | - | - | 3~4 months |
|  | **Sex** | male | male | male | male |
|  | **Species** | SD rat | Wister rat | SD rat | SD rat |
|  | **Location** | China | China | China | Brazil |
|  | **Reference** | Ye et al., 2022 | Dong et al., 2022 | Wang et al., 2021 | Rohden et al., 2021 |

| Supplementary Table S1 (continued) | **Outcome** | ② | ② | ①② | ② | ①② |
| --- | --- | --- | --- | --- | --- | --- |
|  | **Dose** | 200 μl | 4×10^9^ particles | 1×10^11^ particles | 30 μg | 1×10^11^ particles |
|  | **Way of administrated** | Tail vein injection | Striatal transplantation | Tail vein injection | Lateral ventricle injection | Tail vein injection |
|  | **Exosome Diameter** | 30~150 nm | 115.3±6.2 nm | 60~160 nm | 162 nm | 30~100 nm |
|  | **Type of exosomes** | SD rat BMSC-Exos | human NSC-Exos | human iPSC-Exos | Wister rat NSC-Exos | human USC-Exos |
|  | **Group(n)** | MCAO/R+saline(6);  MCAO/R+Exos(6);  MCAO/R+miR-223-3p Exos(6). | MCAO/R+PBS(11);  MCAO/R+Exos(11);  MCAO/R+IFN-γ Exos(11). | MCAO/R+PBS(10);  MCAO/R+Exos(10). | MCAO/R+PBS(8);  MCAO/R+Exos(8). | MCAO/R+PBS(10);  MCAO/R+Exos(10). |
|  | **Age** | - | 8 weeks | 6~8 weeks | 20 months | 6~8 weeks |
|  | **Sex** | male | male | male | male | male |
|  | **Species** | SD rat | SD rat | SD rat | Wister rat | SD rat |
|  | **Location** | China | China | China | Iran | China |
|  | **Reference** | Zhao et al., 2020 | Zhang et al., 2020 | Xia et al., 2020 | Mahdavipour et al., 2020 | Ling et al., 2020 |

| Supplementary Table S1 (continued) | **Outcome** | ② | ① | ② | ② | ①② |
| --- | --- | --- | --- | --- | --- | --- |
|  | **Dose** | 100 μg | 100 μg | 30 μg | 100 μg | 1×10^10^ particles |
|  | **Way of administrated** | Lateral ventricle injection | Tail vein injection | Tail vein injection | Tail vein injection | Intranasal administration |
|  | **Exosome Diameter** | 100 nm | 120 nm | 50~200 nm | 40~150 nm | 50~150 nm |
|  | **Type of exosomes** | SD rat BMSC-Exos | SD rat BMSC-Exos | SD rat BMSC-Exos | ICR mice ADSC-Exos | human iPSC-Exos |
|  | **Group(n)** | MCAO/R+PBS(6);  MCAO/R+Exos(6);  MCAO/R+CXCR4 Exos(6). | MCAO/R+PBS;  MCAO/R+Exos. | MCAO/R+PBS;  MCAO/R+Exos. | MCAO/R+PBS;  MCAO/R+Exos;  MCAO/R+ RA-Exos. | MCAO/R+PBS;  MCAO/R+Exos;  MCAO/R+ BDNF-Exos. |
|  | **Age** | - | 7~8 weeks | 8 weeks | - | 9~10 weeks |
|  | **Sex** | male | male | male | male | male |
|  | **Species** | SD rat | SD rat | SD rat | ICR mice | C57BL/6 mice |
|  | **Location** | China | China | Korea | China | China |
|  | **Reference** | Li et al., 2020 | Han et al., 2020 | Moon et al., 2019 | Liang et al., 2023 | Zhou et al., 2023 |

| Supplementary Table S1 (continued) | **Outcome** | ①② | ① | ①② | ② | ① |
| --- | --- | --- | --- | --- | --- | --- |
|  | **Dose** | 10 μg | 2×10^6^ particles | 50 μg | 2 μg | 1×10^11^ particles |
|  | **Way of administrated** | Lateral ventricle injection | Tail vein injection | Tail vein injection | Intranasal administration | Tail vein injection |
|  | **Exosome Diameter** | <200 nm | 100~150 nm | 30~120 nm | 78.88±16.12 nm | 100~200 nm |
|  | **Type of exosomes** | human NSC-Exos | C57BL/6 mice NPC-Exos, EPC-Exos | C57BL/6 mice BMSC-Exos | C57BL/6 mice ADSC-Exos | human EPC-Exos |
|  | **Group(n)** | MCAO/R+PBS(19);  MCAO/R+Exos(19). | pMCAO+PBS(12);  pMCAO+NPC-Exos(12);  pMCAO+EPC-Exos(12). | MCAO/R+PBS;  MCAO/R+Exos;  MCAO/R+KLF3-AS1 Exos. | MCAO/R+PBS(10);  MCAO/R+Exos(10). | pMCAO+vehicle(20);  pMCAO+Exos(20);  pMCAO+miR-210 Exos(20). |
|  | **Age** | 7~8 weeks | 9.2±0.4 weeks | 8 weeks | 10~12 weeks | 10~12 weeks |
|  | **Sex** | male | male | male | male | male/female |
|  | **Species** | C57BL/6 mice | C57BL/6 mice | C57BL/6 mice | C57BL/6 mice | C57BL/6 mice |
|  | **Location** | China | China | China | China | China |
|  | **Reference** | Zhang et al., 2023 | Xu et al., 2023 | Xie et al., 2023 | Wang et al., 2023b | Wang et al., 2023c |

| Supplementary Table S1 (continued) | **Outcome** | ① | ① | ①② | ① | ① |
| --- | --- | --- | --- | --- | --- | --- |
|  | **Dose** | 1×10^9^ particles | 100 μg | 300 μg | 1×10^9^ particles | 100 μg |
|  | **Way of administrated** | Tail vein injection | Tail vein injection | Tail vein injection | Tail vein injection | Tail vein injection |
|  | **Exosome Diameter** | 30~150 nm | 70±5 nm | 109.23±4.62 nm | 74.7±20.77 nm | 50~150 nm |
|  | **Type of exosomes** | human iPSC-Exos | human BMSC-Exos | C57BL/6 mice NSC-Exos | human iPSC-Exos | C57BL/6 mice BMSC-Exos |
|  | **Group(n)** | MCAO/R+PBS;  MCAO/R+Exos. | MCAO/R+PBS;  MCAO/R+Exos. | MCAO/R+PBS(32);  MCAO/R+Exos(32);  MCAO/R+LBP-Exos(32). | MCAO/R+PBS;  MCAO/R+Exos. | MCAO/R+PBS(3);  MCAO/R+Exos(3);  MCAO/R+3D Exos(3). |
|  | **Age** | 16 months | 2~3 months | - | 16 months | 6~8 weeks |
|  | **Sex** | male | male | male | - | male |
|  | **Species** | C57BL/6 mice | C57BL/6 mice | C57BL/6 mice | C57BL/6 mice | C57BL/6 mice |
|  | **Location** | China | China | China | China | China |
|  | **Reference** | Niu et al., 2023 | Liu et al., 2023 | Li et al., 2023b | Li et al., 2023c | Han et al., 2023 |

| Supplementary Table S1 (continued) | **Outcome** | ①② | ② | ② | ① | ① |
| --- | --- | --- | --- | --- | --- | --- |
|  | **Dose** | 1×10^10^ particles | 800 ng | 100 μg | 50 μg | 50 μg |
|  | **Way of administrated** | Lateral ventricle injection | Lateral ventricle injection | Tail vein injection | Tail vein injection | Tail vein injection |
|  | **Exosome Diameter** | 50~180 nm | 156±7.7 nm | 142±39.4 nm | 108.4 nm | 30~150 nm |
|  | **Type of exosomes** | C57BL/6 mice NSC-Exos | CD1 mice NPC-Exos | ICR mice ADSC-Exos | SD rat BMSC-Exos | human UCMSC-Exos |
|  | **Group(n)** | MCAO/R+PBS;  MCAO/R+Exos;  MCAO/R+HAD-Exos; | MCAO/R+vehicle;  MCAO/R+Exos. | MCAO/R+PBS(14);  MCAO/R+Exos(14). | MCAO/R+PBS;  MCAO/R+Exos. | MCAO/R+PBS;  MCAO/R+Exos;  MCAO/R+miR-146a-5p inhibitor Exos. |
|  | **Age** | 6~8 weeks | 8 weeks | - | 8~10 weeks | 8 weeks |
|  | **Sex** | male | - | male | male | - |
|  | **Species** | C57BL/6 mice | CD1 mice | ICR mice | ICR mice | C57BL/6 mice |
|  | **Location** | China | Mexico | China | China | China |
|  | **Reference** | Gu et al., 2023 | Campero-Romero et al., 2023 | Hu et al., 2022a | Hu et al., 2022b | Zhang et al., 2021 |

| Supplementary Table S1 (continued) | **Outcome** | ① | ①② | ① | ① | ① |
| --- | --- | --- | --- | --- | --- | --- |
|  | **Dose** | 1×10^9^ particles | 10 μg | 1×10^10^ particles | 10 μg | 10 μg |
|  | **Way of administrated** | Tail vein injection | Tail vein injection | Tail vein injection | Femoral vein injection | Jugular injection |
|  | **Exosome Diameter** | 50~150 nm | 100 nm | 100±55 nm | 50~200 nm | - |
|  | **Type of exosomes** | human ESC-Exos | human DPSC-Exos | C57BL/6 mice BMSC-Exos | C57BL/6 mice ADSC-Exos | C57BL/6 mice NSC-Exos |
|  | **Group(n)** | MCAO/R+PBS;  MCAO/R+Exos. | MCAO/R+PBS;  MCAO/R+Exos. | MCAO/R+PBS(10);  MCAO/R+Exos(10);  MCAO/R+miR-132-3p Exos(10). | MCAO/R+PBS(8);  MCAO/R+Exos(8). | MCAO/R+saline;  MCAO/R+Exos. |
|  | **Age** | 8~12 weeks | 10 weeks | 6~8 weeks | 10~12 weeks | - |
|  | **Sex** | male | male | - | male | male |
|  | **Species** | C57BL/6 mice | C57BL/6 mice | C57BL/6 mice | C57BL/6 mice | C57BL/6 mice |
|  | **Location** | China | China | China | Germany | USA |
|  | **Reference** | Xia et al., 2021 | Li et al., 2021 | Pan et al., 2020 | Kuang et al., 2020 | Sun et al., 2019 |

① the cerebral infarct volume (%); ②the mNSS.
