# Supplementary material for: Stem cell-derived exosomes for ischemic stroke: a conventional and network meta-analysis based on animal models
Source: Front Pharmacol. 2024 Oct 23;15:1481617. doi: 10.3389/fphar.2024.1481617 (PMC11537945; doi:10.3389/fphar.2024.1481617)
Supplement: Supplementary file 2 [file Table7.DOCX]

Supplementary Table S7 Network meta-analysis of stem cell-derived exosomes under tail vein injection for the mNSS

| **ADSC-Exos** | -1.32 [-5.68, 2.95] | -0.21 [-6.59, 6.13] | 0.49 [-5.80, 6.83] | 0.56 [-5.84, 7.04] | -0.41 [-7.06, 6.12] | -0.33 [-6.73, 6.04] | 1.66 [-2.08, 5.38] |
| --- | --- | --- | --- | --- | --- | --- | --- |
| 1.32 [-2.95, 5.68] | **BMSC-Exos** | 1.11 [-4.51, 6.81] | 1.83 [-3.74, 7.32] | 1.89 [-3.65, 7.65] | 0.85 [-4.99, 6.90] | 0.96 [-4.60, 6.58] | 2.98 [0.89, 5.18] |
| 0.21 [-6.13, 6.59] | -1.11 [-6.81, 4.51] | **DPSC-Exos** | 0.72 [-6.50, 7.95] | 0.73 [-6.60, 8.23] | -0.26 [-7.73, 7.25] | -0.16 [-7.53, 7.20] | 1.86 [-3.33, 7.06] |
| -0.49 [-6.83, 5.80] | -1.83 [-7.32, 3.74] | -0.72 [-7.95, 6.50] | **NSC-Exos** | 0.04 [-7.10, 7.24] | -0.93 [-8.43, 6.47] | -0.87 [-7.95, 6.46] | 1.17 [-3.82, 6.32] |
| -0.56 [-7.04, 5.84] | -1.89 [-7.65, 3.65] | -0.73 [-8.23, 6.60] | -0.04 [-7.24, 7.10] | **UCMSC-Exos** | -1.00 [-8.59, 6.61] | -0.95 [-8.26, 6.51] | 1.12 [-4.14, 6.30] |
| 0.41 [-6.12, 7.06] | -0.85 [-6.90, 4.99] | 0.26 [-7.25, 7.73] | 0.93 [-6.47, 8.43] | 1.00 [-6.61, 8.59] | **USC-Exos** | 0.06 [-7.47, 7.57] | 2.11 [-3.48, 7.58] |
| 0.33 [-6.04, 6.73] | -0.96 [-6.58, 4.60] | 0.16 [-7.20, 7.53] | 0.87 [-6.46, 7.95] | 0.95 [-6.51, 8.26] | -0.06 [-7.57, 7.47] | **iPSC-Exos** | 2.03 [-3.18, 7.22] |
| -1.66 [-5.38, 2.08] | -2.98 [-5.18, -0.89] | -1.86 [-7.06, 3.33] | -1.17 [-6.32, 3.82] | -1.12 [-6.30, 4.14] | -2.11 [-7.58, 3.48] | -2.03 [-7.22, 3.18] | **Negative control** |
