# Supplementary material for: Stem cell-derived exosomes for ischemic stroke: a conventional and network meta-analysis based on animal models
Source: Front Pharmacol. 2024 Oct 23;15:1481617. doi: 10.3389/fphar.2024.1481617 (PMC11537945; doi:10.3389/fphar.2024.1481617)
Supplement: Supplementary file 3 [file Table2.docx]

| Supplementary Table S2 Characteristics and administration strategies of the exosomes | **Time of detection** | 1 d | 28 d | 1 d | 14 d | 3 d | 28 d | 3 d |
| --- | --- | --- | --- | --- | --- | --- | --- | --- |
|  | **Time-course** | - | 28 d | 1 d | - | 3 d | - | 3 d |
|  | **Frequency** | Only once | Once/1 d | Only once | Only once | Once/1 d | Only noce | Once/1 d |
|  | **Dose** | 200 μg | 100 μg | 1×10^10^ particles | 100 μg | 80 μg | 3×10^11^ particles | 80 μg |
|  | **Timing of administration** | Immediately after surgery | 24 h after surgery | Immediately after surgery | 24 h after surgery | Immediately after surgery | Immediately after surgery | Immediately after surgery |
|  | **Way of administration** | Tail vein injection | Tail vein injection | Tail vein injection | Tail vein injection | Tail vein injection | Tail vein injection | Tail vein injection |
|  | **Immune compatibility** | Allogeneic | Allogeneic | Allogeneic | Allogeneic | Xenogeneic | Xenogeneic | Xenogeneic |
|  | **Isolation and purification** | Exosome isolation kit | Ultracentrifugation | Exosome isolation kit | Exosome isolation kit | Ultracentrifugation | Ultracentrifugation | Ultracentrifugation |
|  | **Type of exosomes** | SD rat BMSC-Exos | SD rat NSC-Exos | SD rat BMSC-Exos | SD rat BMSC-Exos | human UCMSC-Exos | human BMSC-Exos | human UCMSC-Exos |
|  | **Reference** | Wang et al., 2023a | Long et al., 2023 | Li et al., 2023a | Jiang et al., 2023 | Ye et al., 2022 | Dong et al., 2022 | Wang et al., 2021 |

| Supplementary Table S2 (continued) | **Time of detection** | 2 d | 28 d | 28 d | 28 d | 7 d | 28 d | 21 d |
| --- | --- | --- | --- | --- | --- | --- | --- | --- |
|  | **Time-course** | - | 28 d | - | - | - | - | - |
|  | **Frequency** | Only once | Once/2 w | Only once | Only once | Only once | Only once | Only once |
|  | **Dose** | 200 μg/Kg | 200 μl | 4×10^9^ particles | 1×10^11^ particles | 30 μg | 1×10^11^ particles | 100 μg |
|  | **Timing of administration** | 24 h after surgery | 24 h after surgery | 24 h after surgery | 4 h after surgery | 2 h after surgery | 4 h after surgery | 24 h after surgery |
|  | **Way of administration** | Intranasal administration | Tail vein injection | Sriatal transplantation | Tail vein injectio | Lateral ventricle injection | Tail vein injectio | Lateral ventricle injection |
|  | **Immune compatibility** | Xenogeneic | Allogeneic | Xenogeneic | Xenogeneic | Allogeneic | Xenogeneic | Allogeneic |
|  | **Isolation and purification** | Ultracentrifugation | Ultracentrifugation | Ultracentrifugation | Ultracentrifugation | Ultracentrifugation | Ultracentrifugation | Ultracentrifugation |
|  | **Type of exosomes** | human ADSC-Exos | SD rat BMSC-Exos | human NSC-Exos | iPSC-Exos | Wister rat NSC-Exos | human USC-Exos | SD rat BMSC-Exos |
|  | **Reference** | Rohden et al., 2021 | Zhao et al., 2020 | Zhang et al., 2020 | Xia et al., 2020 | Mahdavipour et al., 2020 | Ling et al., 2020 | Li et al., 2020 |

| Supplementary Table S2 (continued) | **Time of detection** | 2 d | 16 d | 3 d | 3 d | 8 w | 1 d | 28 d |
| --- | --- | --- | --- | --- | --- | --- | --- | --- |
|  | **Time-course** | - | - | 3 d | 3 d | - | - | 28 d |
|  | **Frequency** | - | Only once | Once/1 d | Once/1 d | Only once | Only once | Once/2 w |
|  | **Dose** | 100 μg | 30 μg | 100 μg | 1×10^10^ particles | 10 μg | 2×10^6^ particles | 50 μg |
|  | **Timing of administration** | Immediately after surgery | 24 h after surgery | 6 h after surgery | 2 h after surgery | 7 d after surgery | 2 h after surgery | 24 h after surgery |
|  | **Way of administration** | Tail vein injectio | Tail vein injection | Tail vein injection | Intranasal administration | Lateral ventricle injection | Tail vein injection | Tail vein injection |
|  | **Immune compatibility** | Allogeneic | Allogeneic | Allogeneic | Xenogeneic | Xenogeneic | Allogeneic | Allogeneic |
|  | **Isolation and purification** | Ultracentrifugation | Ultracentrifugation | Ultracentrifugation | Anion exchange | Ultracentrifugation | Ultracentrifugation | Ultracentrifugation |
|  | **Type of exosomes** | SD rat BMSC-Exos | SD rat BMSC-Exos | ICR mice ADSC-Exos | iPSC-Exos | human NSC-Exos | C57BL/6 mice NPC-Exos, EPC-Exos | C57BL/6 mice BMSC-Exos |
|  | **Reference** | Han et al., 2020 | Moon et al., 2019 | Liang et al., 2023 | Zhou et al., 2023 | Zhang et al., 2023 | Xu et al., 2023 | Xie et al., 2023 |

| Supplementary Table S2 (continued) | **Time of detection** | 28 d | 1 d | 3 d | 1 d | 28 d | 3 d | 3 d |
| --- | --- | --- | --- | --- | --- | --- | --- | --- |
|  | **Time-course** | 28 d | - | - | - | 3 d | - | 3 d |
|  | **Frequency** | Once/1 d | Only once | Only once | Only once | Twice/1 d | Only once | Once/1 d |
|  | **Dose** | 10 μg | 1×10^11^ particles | 2×10^9^ particles | 100 μg | 300 μg | 1×10^9^ particles | 100 μg |
|  | **Timing of administration** | 24 h after surgery | 2 h after surgery | 2 months befor surgery | 0.5 h after surgery | 6 h after surgery | 2 months before surgery | - |
|  | **Way of administration** | Intranasal administration | Tail vein injection | Tail vein injection | Tail vein injection | Tail vein injection | Tail vein injection | Tail vein injection |
|  | **Immune compatibility** | Allogeneic | Xenogeneic | Xenogeneic | Xenogeneic | Allogeneic | Xenogeneic | Allogeneic |
|  | **Isolation and purification** | Ultracentrifugation | Ultracentrifugation | Ultracentrifugation | Ultracentrifugation | Ultracentrifugation | Ultracentrifugation | Ultracentrifugation |
|  | **Type of exosomes** | C57BL/6 mice ADSC-Exos | human EPC-Exos | iPSC-Exos | human BMSC-Exos | C57BL/6 mice NSC-Exos | iPSC-Exos | C57BL/6 mice BMSC-Exos |
|  | **Reference** | Wang et al., 2023b | Wang et al., 2023c | Niu et al., 2023 | Liu et al., 2023 | Li et al., 2023b | Li et al., 2023c | Han et al., 2023 |

| Supplementary Table S2 (continued) | **Time of detection** | 28 d | 14 d | 14 d | 14 d | 3 d | 7 d | 7 d |
| --- | --- | --- | --- | --- | --- | --- | --- | --- |
|  | **Time-course** | - | 2 d | 7 d | - | - | 7 d | - |
|  | **Frequency** | Only once | Once/1 d | Once/1 d | Only once | Only once | 3 times/1 d | Only once |
|  | **Dose** | 1×10^10^ particles | 800 ng | 100 μg | 50 μg | 50 μg | 1×10^9^ particles | 10 μg |
|  | **Timing of administration** | Immediately after surgery | 0.5 h after surgery | 24 h after surgery | 24 h after surgery | 4 h after surgery | 2 h after surgery | Immediately after surgery |
|  | **Way of administration** | Lateral ventricle injection | Lateral ventricle injection | Tail vein injection | Tail vein injection | Tail vein injection | Tail vein injection | Tail vein injection |
|  | **Immune compatibility** | Allogeneic | Allogeneic | Allogeneic | Xenogeneic | Xenogeneic | Xenogeneic | Xenogeneic |
|  | **Isolation and purification** | Ultracentrifugation | Ultracentrifugation | Ultracentrifugation | Ultracentrifugation | Ultracentrifugation | Ultracentrifugation | Exosome isolation kit |
|  | **Type of exosomes** | C57BL/6 mice NSC-Exos | CD1 mice NPC-Exos | ICR mice ADSC-Exos | SD rat BMSC-Exos | human UCMSC-Exos | human ESC-Exos | human DPSC-Exos |
|  | **Reference** | Gu et al., 2023 | Campero-Romero et al., 2023 | Hu et al., 2022a | Hu et al., 2022b | Zhang et al., 2021 | Xia et al., 2021 | Li et al., 2021 |

| Supplementary Table S2 (continued) | **Time of detection** | 2 d | 2 d | 4 d |
| --- | --- | --- | --- | --- |
|  | **Time-course** | - | - | - |
|  | **Frequency** | Only once | Only once | Only once |
|  | **Dose** | 1×10^10^ particles | 10 μg | 10 μg |
|  | **Timing of administration** | Immediately after surgery | Immediately after surgery | 2 h after surgery |
|  | **Way of administration** | Tail vein injection | Femoral vein injection | Jugular vein injection |
|  | **Immune compatibility** | Allogeneic | Allogeneic | Allogeneic |
|  | **Isolation and purification** | Ultracentrifugation | Polyethylene glycol precipitation | Polyethylene glycol precipitation |
|  | **Type of exosomes** | C57BL/6 mice BMSC-Exos | C57BL/6 mince ADSC-Exos | C57BL/6 mince NSC-Exos |
|  | **Reference** | Pan et al., 2020 | Kuang et al., 2020 | Sun et al., 2019 |
