# Supplementary material for: Stem cell-derived exosomes for ischemic stroke: a conventional and network meta-analysis based on animal models
Source: Front Pharmacol. 2024 Oct 23;15:1481617. doi: 10.3389/fphar.2024.1481617 (PMC11537945; doi:10.3389/fphar.2024.1481617)
Supplement: Supplementary file 4 [file Table5.DOCX]

Supplementary Table S5 Network meta-analysis of stem cell-derived exosomes under tail vein injection for the cerebral infarct volume (%)

| **ADSC-Exos** | -12.63 [-32.57, 7.61] | -8.37 [-35.51, 18.49] | -0.96 [-23.61, 21.10] | 0.50 [-27.29, 27.75] | -4.28 [-28.34, 19.65] | -2.09 [-24.58, 19.74] | 1.41 [-20.44, 23.03] | 1.45 [-26.91, 31.66] | -8.55 [-30.07, 13.55] | 10.75 [-8.34, 29.84] |
| --- | --- | --- | --- | --- | --- | --- | --- | --- | --- | --- |
| 12.63 [-7.61, 32.57] | **BMSC-Exos** | 4.29 [-15.09, 23.41] | 11.45 [-1.59, 24.59] | 13.09 [-7.48, 33.13] | 8.43 [-7.53, 24.21] | 10.27 [-2.01, 23.01] | 13.96 [1.65, 26.10] | 14.41 [-8.93, 37.75] | 4.08 [-8.03, 16.27] | 23.28 [17.33, 29.43] |
| 8.37 [-18.49, 35.51] | -4.29 [-23.41, 15.09] | **DPSC-Exos** | 7.30 [-14.13, 29.11] | 8.91 [-18.44, 35.41] | 4.44 [-19.77, 27.80] | 6.11 [-15.53, 27.60] | 9.61 [-11.12, 30.56] | 10.10 [-19.34, 39.71] | -0.08 [-21.69, 21.36] | 19.13 [0.65, 37.39] |
| 0.96 [-21.10, 23.61] | -11.45 [-24.59, 1.59] | -7.30 [-29.11, 14.13] | **EPC-Exos** | 1.59 [-21.83, 24.44] | -3.02 [-17.71, 11.45] | -1.26 [-17.25, 15.14] | 2.41 [-13.46, 18.44] | 2.80 [-22.11, 27.91] | -7.41 [-23.62, 8.67] | 11.83 [-0.13, 23.61] |
| -0.50 [-27.75, 27.29] | -13.09 [-33.13, 7.48] | -8.91 [-35.41, 18.44] | -1.59 [-24.44, 21.83] | **ESC-Exos** | -4.62 [-29.29, 20.30] | -2.80 [-24.73, 19.57] | 0.81 [-21.03, 23.26] | 1.29 [-27.73, 31.25] | -8.92 [-31.24, 13.22] | 10.20 [-9.36, 30.28] |
| 4.28 [-19.65, 28.34] | -8.43 [-24.21, 7.53] | -4.44 [-27.80, 19.77] | 3.02 [-11.45, 17.71] | 4.62 [-20.30, 29.29] | **NPC-Exos** | 1.82 [-16.70, 20.25] | 5.32 [-12.90, 23.62] | 5.81 [-20.69, 32.58] | -4.28 [-22.97, 14.03] | 14.91 [-0.15, 29.60] |
| 2.09 [-19.74, 24.58] | -10.27 [-23.01, 2.01] | -6.11 [-27.60, 15.53] | 1.26 [-15.14, 17.25] | 2.80 [-19.57, 24.73] | -1.82 [-20.25, 16.70] | **NSC-Exos** | 3.50 [-11.49, 18.49] | 4.04 [-20.83, 28.83] | -6.15 [-21.59, 9.11] | 13.08 [1.85, 23.97] |
| -1.41 [-23.03, 20.44] | **-13.96 [-26.10, -1.65]** | -9.61 [-30.56, 11.12] | -2.41 [-18.44, 13.46] | -0.81 [-23.26, 21.03] | -5.32 [-23.62, 12.90] | -3.50 [-18.49, 11.49] | **UCMSC-Exos** | 0.45 [-24.17, 25.06] | -9.70 [-25.37, 4.76] | 9.46 [-0.84, 19.84] |
| -1.45 [-31.66, 26.91] | -14.41 [-37.75, 8.93] | -10.10 [-39.71, 19.34] | -2.80 [-27.91, 22.11] | -1.29 [-31.25, 27.73] | -5.81 [-32.58, 20.69] | -4.04 [-28.83, 20.83] | -0.45 [-25.06, 24.17] | **USC-Exos** | -10.29 [-34.65, 13.92] | 9.00 [-13.29, 31.03] |
| 8.55 [-13.55, 30.07] | -4.08 [-16.27, 8.03] | 0.08 [-21.36, 21.69] | 7.41 [-8.67, 23.62] | 8.92 [-13.22, 31.24] | 4.28 [-14.03, 22.97] | 6.15 [-9.11, 21.59] | 9.70 [-4.76, 25.37] | 10.29 [-13.92, 34.65] | **iPSC-Exos** | 19.27 [8.33, 30.01] |
| -10.75 [-29.84, 8.34] | -23.28 [-29.43, -17.33] | -19.13 [-37.39, -0.65] | -11.83 [-23.61, 0.13] | -10.20 [-30.28, 9.36] | -14.91 [-29.60, 0.15] | -13.08 [-23.97, -1.85] | -9.46 [-19.84, 0.84] | -9.00 [-31.03, 13.29] | -19.27 [-30.01, -8.33] | **Negative control** |

Significant results were bolded.
