# Supplementary material for: Stem cell-derived exosomes for ischemic stroke: a conventional and network meta-analysis based on animal models
Source: Front Pharmacol. 2024 Oct 23;15:1481617. doi: 10.3389/fphar.2024.1481617 (PMC11537945; doi:10.3389/fphar.2024.1481617)
Supplement: Supplementary file 5 [file Table3.DOCX]

**Supplementary Table S3 Subgroup analysis results for the cerebral infarct volume (%)**

| **Routes of administration** | **Mice model**  **(Number of studies =18)** | | **Rat model**  **(Number of studies =11)** | |
| --- | --- | --- | --- | --- |
|  | **SMD** | **95% CI** | **SMD** | **95% CI** |
| Intracerebral administration vs. Intranasal administration | 7.39 | [-13.64, 29.28] | - | - |
| Intracerebral administration vs. Intravenous administration | 9.52 | [-3.71, 24.01] | - | - |
| Intracerebral administration vs. Negative control | -6.94 | [-19.45, 6.51] | - | - |
| Intranasal administration vs. Intravenous administration | 2.15 | [-15.58, 20.01] | 11.12 | [-15.23, 37.52] |
| Intranasal administration vs. Negative control | -14.33 | [-31.38, 2.90] | -6.41 | [-32.04, 18.79] |
| Intravenous administration vs. Negative control | -16.48 | [-21.00, -12.03] | -17.65 | [-25.87, -9.58] |
| **Types of stem cell-derived exosomes under intravenous administration** | | | | |
| ADSC-Exos vs. BMSC-Exos | 13.02 | [-7.17, 33.32] | - | - |
| ADSC-Exos vs. DPSC-Exos | 8.19 | [-16.79, 33.28] | - | - |
| ADSC-Exos vs. EPC-Exos | 0.64 | [-20.21, 22.48] | - | - |
| ADSC-Exos vs. ESC-Exos | -0.58 | [-25.93, 25.62] | - | - |
| ADSC-Exos vs. NPC-Exos | 3.74 | [-18.94, 27.27] | - | - |
| ADSC-Exos vs. NSC-Exos | -0.92 | [-23.35, 21.99] | - | - |
| ADSC-Exos vs. UCMSC-Exos | -1.95 | [-27.03, 24.44] | - | - |
| ADSC-Exos vs. iPSC-Exos | 10.81 | [-10.66, 33.60] | - | - |
| ADSC-Exos vs. Negative control | -10.71 | [-28.97, 7.57] | - | - |
| BMSC-Exos vs. DPSC-Exos | -4.86 | [-24.87, 14.39] | - | - |
| BMSC-Exos vs. EPC-Exos | -12.58 | [-25.74, 2.08] | - | - |
| BMSC-Exos vs. ESC-Exos | -13.56 | [-34.07, 7.02] | - | - |
| BMSC-Exos vs. NPC-Exos | -9.41 | [-25.48, 7.97] | - | - |
| BMSC-Exos vs. NSC-Exos | -13.91 | [-29.66, 1.78] | -4.54 | [-41.54, 31.85] |
| BMSC-Exos vs. UCMSC-Exos | -15.00 | [-34.57, 5.51] | -13.13 | [-40.74, 15.85] |
| BMSC-Exos vs. iPSC-Exos | -2.44 | [-16.79, 13.57] | -7.73 | [-44.07, 29.04] |
| BMSC-Exos vs. Negative control | -23.81 | [-32.65, -15.39] | -22.73 | [-37.54, -7.68] |
| DPSC-Exos vs. EPC-Exos | -7.60 | [-28.13, 13.77] | - | - |
| DPSC-Exos vs. ESC-Exos | -8.84 | [-34.18, 17.27] | - | - |
| DPSC-Exos vs. NPC-Exos | -4.57 | [-26.67, 18.70] | - | - |
| DPSC-Exos vs. NSC-Exos | -9.12 | [-30.49, 12.86] | - | - |
| DPSC-Exos vs. UCMSC-Exos | -10.07 | [-35.44, 15.85] | - | - |
| DPSC-Exos vs. iPSC-Exos | 2.44 | [-18.44, 25.22] | - | - |
| DPSC-Exos vs. Negative control | -18.92 | [-36.14, -1.35] | - | - |
| EPC-Exos vs. ESC-Exos | -1.20 | [-23.26, 20.25] | - | - |
| EPC-Exos vs. NPC-Exos | 3.16 | [-10.75, 16.59] | - | - |
| EPC-Exos vs. NSC-Exos | -1.50 | [-18.74, 15.48] | - | - |
| EPC-Exos vs. UCMSC-Exos | -2.25 | [-24.08, 19.23] | - | - |
| EPC-Exos vs. iPSC-Exos | 10.22 | [-6.47, 26.92] | - | - |
| EPC-Exos vs. Negative control | -11.31 | [-23.23, -0.81] | - | - |
| ESC-Exos vs. NPC-Exos | 4.26 | [-18.64, 27.66] | - | - |
| ESC-Exos vs. NSC-Exos | -0.22 | [-23.60, 22.43] | - | - |
| ESC-Exos vs. UCMSC-Exos | -1.25 | [-27.33, 24.64] | - | - |
| ESC-Exos vs. iPSC-Exos | 11.21 | [-11.10, 34.26] | - | - |
| ESC-Exos vs. Negative control | -10.14 | [-29.19, 8.03] | - | - |
| NPC-Exos vs. NSC-Exos | -4.56 | [-24.12, 14.65] | - | - |
| NPC-Exos vs. UCMSC-Exos | -5.52 | [-29.25, 17.30] | - | - |
| NPC-Exos vs. iPSC-Exos | 7.01 | [-11.61, 26.16] | - | - |
| NPC-Exos vs. Negative control | -14.46 | [-29.35, -0.68] | - | - |
| NSC-Exos vs. UCMSC-Exos | -0.93 | [-23.15, 21.50] | -8.63 | [-50.08, 31.87] |
| NSC-Exos vs. iPSC-Exos | 11.73 | [-6.58, 30.14] | -3.49 | [-50.08, 31.87] |
| NSC-Exos vs. Negative control | -9.84 | [-22.96, 3.04] | -18.31 | [-51.72, 15.11] |
| UCMSC-Exos vs. iPSC-Exos | 12.56 | [-9.72, 35.49] | 5.19 | [-34.99, 46.15] |
| UCMSC-Exos vs. Negative control | -8.96 | [-27.75, 9.30] | -9.63 | [-33.04, 14.19] |
| iPSC-Exos vs. Negative control | -21.48 | [-34.87, -9.49] | -14.94 | [-49.14, 18.32] |
| USC-Exos vs. BMSC-Exos | - | - | 13.70 | [-25.45, 52.37] |
| USC-Exos vs. NSC-Exos | - | - | 9.33 | [-41.05, 57.37] |
| USC-Exos vs. UCMSC-Exos | - | - | 0.48 | [-42.19, 43.79] |
| USC-Exos vs. iPSC-Exos | - | - | 5.89 | [-42.94, 55.43] |
| USC-Exos vs. Negative control | - | - | -8.95 | [-45.54, 26.03] |
| **Immune compatibility of stem cell-derived exosomes under intravenous administration** | | | | |
| Allogeneic vs. Xenogeneic | 2.53 | [-6.60, 12.17] | -5.29 | [-22.72, 12.1] |
| Allogeneic vs. Negative control | -15.29 | [-21.94, -8.74] | -20.12 | [-32.39, -8.07] |
| Xenogeneic vs. Negative control | -17.79 | [-24.88, -11.45] | -14.80 | [-27.18, -2.55] |
