# Supplementary material for: Stem cell-derived exosomes for ischemic stroke: a conventional and network meta-analysis based on animal models
Source: Front Pharmacol. 2024 Oct 23;15:1481617. doi: 10.3389/fphar.2024.1481617 (PMC11537945; doi:10.3389/fphar.2024.1481617)
Supplement: Supplementary file 6 [file Table4.DOCX]

| Supplementary Table S4 Node splitting test | | | | | | | |
| --- | --- | --- | --- | --- | --- | --- | --- |
| Side | Direct | | Indirect | | Difference | | |
|  | Coef. | Std.Err. | Coef. | Std.Err. | Coef. | Std.Err. | *P* |
| ADSC-Exos vs. Negative control | 1.39 | 1.11 | 1.22 | 6.21 | 0.17 | 6.30 | 0.98 |
| BMSC-Exos vs. Negative control | 2.57 | 0.52 | 2.76 | 20.13 | -0.19 | 20.13 | 0.99 |
| DPSC-Exos vs. Negative control | 3.56 | 1.45 | 2.76 | 63.31 | 0.80 | 63.32 | 0.99 |
| EPC-Exos vs. Negative control | 2.04 | 0.86 | 1.48 | 1.61 | 0.56 | 1.83 | 0.76 |
| NPC-Exos vs. Negative control | 1.60 | 1.15 | 2.16 | 1.42 | -0.56 | 1.83 | 0.76 |
| EPC-Exos vs. NPC-Exos | -0.13 | 1.13 | 0.43 | 1.44 | -0.56 | 1.83 | 0.76 |
| ESC-Exos vs. Negative control | 1.09 | 1.09 | 3.01 | 63.31 | -1.91 | 63.31 | 0.98 |
| iPSC-Exos vs. Negative control | 3.66 | 0.80 | 2.76 | 36.59 | 0.90 | 36.60 | 0.98 |
| NSC-Exos vs. Negative control | 1.83 | 0.70 | 2.78 | 36.59 | -0.95 | 36.60 | 0.98 |
| UCMSC-Exos vs. Negative control | 2.56 | 0.92 | 2.76 | 36.59 | -0.51 | 36.61 | 0.99 |
| USC-Exos vs. Negative control | 0.57 | 1.05 | 2.76 | 63.31 | -2.19 | 63.32 | 0.97 |
