# Supplementary material for: Stem cell-derived exosomes for ischemic stroke: a conventional and network meta-analysis based on animal models
Source: Front Pharmacol. 2024 Oct 23;15:1481617. doi: 10.3389/fphar.2024.1481617 (PMC11537945; doi:10.3389/fphar.2024.1481617)
Supplement: Supplementary file 7 [file Table6.DOCX]

**Supplementary Table S6 Subgroup analysis results for the mNSS**

| **Routes of administration** | **Mice model**  **(Number of studies =10)** | | **Rat model**  **(Number of studies =11)** | |
| --- | --- | --- | --- | --- |
|  | **SMD** | **95% CI** | **SMD** | **95% CI** |
| Intracerebral administration vs. Intranasal administration | 0.50 | [-1.32, 2.20] | - | - |
| Intracerebral administration vs. Intravenous administration | 0.95 | [-0.50, 2.58] | 1.55 | [-1.30, 4.29] |
| Intracerebral administration vs. Negative control | -0.69 | [-2.04, 0.62] | -0.88 | [-3.25, 1.44] |
| Intranasal administration vs. Intravenous administration | 0.42 | [-0.78, 2.09] | - | - |
| Intranasal administration vs. Negative control | -1.20 | [-2.31, 0.06] | - | - |
| Intravenous administration vs. Negative control | -1.62 | [-2.60, -1.00] | -2.44 | [-3.91, -1.01] |
| **Types of stem cell-derived exosomes under intravenous administration** | | | | |
| ADSC-Exos vs. BMSC-Exos | 1.94 | [-3.15, 6.58] | - | - |
| ADSC-Exos vs. DPSC-Exos | 0.22 | [-4.61, 4.84] | - | - |
| ADSC-Exos vs. NSC-Exos | -0.47 | [-5.32, 4.06] | - | - |
| ADSC-Exos vs. Negative control | -1.64 | [-4.45, 0.96] | - | - |
| BMSC-Exos vs. DPSC-Exos | -1.70 | [-7.01, 4.10] | - | - |
| BMSC-Exos vs. NSC-Exos | -2.41 | [-7.80, 3.19] | - | - |
| BMSC-Exos vs. UCMSC-Exos | - | - | -1.78 | [-9.47, 5.87] |
| BMSC-Exos vs. USC-Exos | - | - | -0.81 | [-8.28, 6.7] |
| BMSC-Exos vs. iPSC-Exos | - | - | -0.81 | [-8.16, 6.43] |
| BMSC-Exos vs. Negative control | -3.56 | [-7.46, 0.55] | -2.89 | [-6.03, 0.20] |
| DPSC-Exos vs. NSC-Exos | -0.68 | [-6.29, 4.72] | - | - |
| DPSC-Exos vs. Negative control | -1.85 | [-5.77, 2.01] | - | - |
| NSC-Exos vs. Negative control | -1.16 | [-4.86, 2.71] | - | - |
| UCMSC-Exos vs. USC-Exos | - | - | 1.00 | [-8.64, 10.89] |
| UCMSC-Exos vs. iPSC-Exos | - | - | 0.98 | [-8.75, 10.83] |
| UCMSC-Exos vs. Negative control | - | - | -1.10 | [-8.04, 5.93] |
| USC-Exos vs. iPSC-Exos | - | - | -0.06 | [-9.75, 9.7] |
| USC-Exos vs. Negative control | - | - | -2.08 | [-9.04, 4.72] |
| iPSC-Exos vs. Negative control | - | - | -2.05 | [-8.85, 4.60] |
| **Immune compatibility of stem cell-derived exosomes under intravenous administration** | | | | |
| Allogeneic vs. Xenogeneic | 0.06 | [-3.65, 3.44] | 0.91 | [-2.95, 4.58] |
| Allogeneic vs. Negative control | -1.73 | [-3.56, -0.34] | -1.97 | [-4.70, 0.67] |
| Xenogeneic vs. Negative control | -1.84 | [-5.03, 1.30] | -2.90 | [-5.60, -0.13] |
