# Supplementary material for: Stem cell-derived exosomes for ischemic stroke: a conventional and network meta-analysis based on animal models
Source: Front Pharmacol. 2024 Oct 23;15:1481617. doi: 10.3389/fphar.2024.1481617 (PMC11537945; doi:10.3389/fphar.2024.1481617)
Supplement: Supplementary file 8 [file DataSheet1.docx]

**Supplementary Material S1 Detailed search strategies for each database**

**1 Pubmed (183)**

((Ischemic Stroke[MeSH Terms]) OR (Ischemic Stroke[MeSH Major Topic]) OR (Ischemic Stroke*[Title/Abstract]) OR (Ischaemic Stroke*[Title/Abstract]) OR (Cryptogenic Ischemic Stroke*[Title/Abstract]) OR (Cryptogenic Stroke*[Title/Abstract]) OR (Cryptogenic Embolism Stroke*[Title/Abstract]) OR (Wake-up Stroke*[Title/Abstract]) OR (Wake up Stroke*[Title/Abstract]) OR (Acute Ischemic Stroke*[Title/Abstract]) OR (Cerebral Infarction[MeSH Terms]) OR (Cerebral Infarction[MeSH Major Topic]) OR (Cerebral Infarct*[Title/Abstract]) OR (Left Hemisphere, Infarction, Cerebral[Title/Abstract]) OR (Infarction, Left Hemisphere, Cerebral[Title/Abstract]) OR (Cerebral, Left Hemisphere, Infarction[Title/Abstract]) OR (Infarction, Cerebral, Left Hemisphere[Title/Abstract]) OR (Subcortical Infarction[Title/Abstract]) OR (Infarction, Subcortical[Title/Abstract]) OR (Subcortical Infarctions[Title/Abstract]) OR (Posterior Choroidal Artery Infarction[Title/Abstract]) OR (Anterior Choroidal Artery Infarction[Title/Abstract]) OR (Infarction, Right Hemisphere, Cerebral[Title/Abstract]) OR (Right Hemisphere, Infarction, Cerebral[Title/Abstract]) OR (Cerebral, Right Hemisphere, Infarction[Title/Abstract]) OR (Infarction, Cerebral, Right Hemisphere[Title/Abstract]) OR (Brain Infarction[MeSH Terms]) OR (Brain Infarction[MeSH Major Topic]) OR (Infarction, Brain[Title/Abstract]) OR (Brain Infarct*[Title/Abstract]) OR (Infarct, Brain[Title/Abstract]) OR (Anterior Circulation Brain Infarction[Title/Abstract]) OR (Infarction, Brain, Anterior Circulation[Title/Abstract]) OR (Infarction, Anterior Circulation, Brain[Title/Abstract]) OR (Anterior Circulation Infarction, Brain[Title/Abstract]) OR (Venous Infarction, Brain[Title/Abstract]) OR (Brain Venous Infarction*[Title/Abstract]) OR (Infarction, Brain Venous[Title/Abstract]) OR (Venous Infarctions, Brain[Title/Abstract]) OR (Infarction, Venous Brain[Title/Abstract]) OR (Infarctions, Venous Brain[Title/Abstract]) OR (Anterior Cerebral Circulation Infarction[Title/Abstract]) OR (Infarction, Anterior Cerebral Circulation[Title/Abstract]) OR (Posterior Circulation Infarction, Brain[Title/Abstract]) OR (Infarction, Brain, Posterior Circulation[Title/Abstract]) OR (Infarction, Posterior Circulation, Brain[Title/Abstract])) **AND** ((stem cells[MeSH Terms]) OR (stem cells[MeSH Major Topic]) OR (Stem Cell[Title/Abstract]) OR (Progenitor Cell*[Title/Abstract]) OR (Mother Cell*[Title/Abstract]) OR (Mesenchymal Stem Cells[MeSH Terms]) OR (Mesenchymal Stem Cells[MeSH Major Topic]) OR (Bone Marrow Stromal Cell*[Title/Abstract]) OR (Multipotent Bone Marrow Stromal Cell*[Title/Abstract]) OR (Adipose-Derived Mesenchymal Stromal Cells[Title/Abstract]) OR (Adipose Derived Mesenchymal Stromal Cells[Title/Abstract]) OR (Adipose Tissue-Derived Mesenchymal Stromal Cell*[Title/Abstract]) OR (Adipose Tissue Derived Mesenchymal Stromal Cell*[Title/Abstract]) OR (Mesenchymal Stromal Cell*[Title/Abstract]) OR (Multipotent Mesenchymal Stromal Cell*[Title/Abstract]) OR (Mesenchymal Progenitor Cell*[Title/Abstract]) OR (Neural Stem Cells[MeSH Terms]) OR (Neural Stem Cells[MeSH Major Topic]) OR (Neural Stem Cell[Title/Abstract]) OR (Induced Pluripotent Stem Cells[MeSH Terms]) OR (Induced Pluripotent Stem Cells[MeSH Major Topic]) OR (IPS Cell*[Title/Abstract]) OR (hiPSC[Title/Abstract]) OR (Adult Stem Cells[MeSH Terms]) OR (Adult Stem Cells[MeSH Major Topic]) OR (Adult Stem Cell[Title/Abstract]) OR (Somatic Stem Cell*[Title/Abstract]) OR (Fetal Stem Cells[MeSH Terms]) OR (Fetal Stem Cells[MeSH Major Topic]) OR (Embryonic Stem Cells[MeSH Major Topic]) OR (Embryonic Stem Cells[MeSH Terms]) OR (Multipotent Stem Cells[MeSH Terms]) OR (Multipotent Stem Cells[MeSH Major Topic]) OR (Multipotent Stem Cell[Title/Abstract]) OR (Totipotent Stem Cell[Title/Abstract]) OR (Totipotent Stem Cells[MeSH Terms]) OR (Totipotent Stem Cells[MeSH Major Topic]) OR (Hematopoietic Stem Cells[MeSH Major Topic]) OR (Hematopoietic Stem Cells[MeSH Terms]) OR (Hematopoietic Stem Cell[Title/Abstract]) OR (Hematopoietic Progenitor Cell*[Title/Abstract]) OR (Hematopoietic Colony-Forming Unit*[Title/Abstract]) OR (Peripheral Blood Stem Cells[MeSH Terms]) OR (Peripheral Blood Stem Cells[MeSH Major Topic]) OR (Erythroid Precursor Cells[MeSH Major Topic]) OR (Erythroid Precursor Cells[MeSH Terms]) OR (Erythroid Colony-Forming Unit*[Title/Abstract]) OR (Erythroid Progenitor Cell*[Title/Abstract]) OR (Erythroid Stem Cell*[Title/Abstract]) OR (Erythropoietic Stem Cell*[Title/Abstract]) OR (Erythropoietic Progenitor Cell*[Title/Abstract]) OR (Erythroid Burst-Forming Unit*[Title/Abstract])) **AND** ((Exosomes[MeSH Terms]) OR (Exosomes[MeSH Major Topic]) OR (exosome*[Title/Abstract]) OR (extracellular vesicle*[Title/Abstract]) OR (Secretory Vesicle*[Title/Abstract]) OR (Cell-Derived Microparticle*[Title/Abstract]) OR (secretome*[Title/Abstract]) OR (membrane vesicle*[Title/Abstract]) OR (Extracellular Vesicles[MeSH Terms]) OR (Extracellular Vesicles[MeSH Major Topic]) OR (Extracellular Vesicle*[Title/Abstract]) OR (Exovesicle*[Title/Abstract]) OR (Apoptotic Bod*[Title/Abstract]))

**2 Web of Science (371)**

#1

TS=(“Ischemic Stroke” OR “Ischaemic Stroke*” OR “Cryptogenic Ischemic Stroke*” OR “Cryptogenic Stroke*” OR “Cryptogenic Embolism Stroke*” OR “Wake-up Stroke*” OR “Wake up Stroke*” OR “Acute Ischemic Stroke*” OR “Cerebral Infarction” OR “Cerebral Infarct*” OR “Left Hemisphere, Infarction, Cerebral” OR “Infarction, Left Hemisphere, Cerebral” OR “Cerebral, Left Hemisphere, Infarction” OR “Infarction, Cerebral, Left Hemisphere” OR “Subcortical Infarction” OR “Infarction, Subcortical” OR “Subcortical Infarctions” OR “Posterior Choroidal Artery Infarction” OR “Anterior Choroidal Artery Infarction” OR “Infarction, Right Hemisphere, Cerebral” OR “Right Hemisphere, Infarction, Cerebral” OR “Cerebral, Right Hemisphere, Infarction” OR “Infarction, Cerebral, Right Hemisphere”)

#2

TS=(“Brain Infarction” OR “Infarction, Brain” OR “Brain Infarct*” OR “Infarct, Brain” OR “Anterior Circulation Brain Infarction” OR “Infarction, Brain, Anterior Circulation” OR “Infarction, Anterior Circulation, Brain” OR “Anterior Circulation Infarction, Brain” OR “Venous Infarction, Brain” OR “Brain Venous Infarction*” OR “Infarction, Brain Venous” OR “Venous Infarctions, Brain” OR “Infarction, Venous Brain” OR “Infarctions, Venous Brain” OR “Anterior Cerebral Circulation Infarction” OR “Infarction, Anterior Cerebral Circulation” OR “Posterior Circulation Infarction, Brain” OR “Infarction, Brain, Posterior Circulation” OR “Infarction, Posterior Circulation, Brain”)

**#3=#1 OR #2**

#4

TS=(“stem cell*” OR “Progenitor Cell*” OR “Mother Cell*” OR “Mesenchymal Stem Cells” OR “Bone Marrow Stromal Cell*” OR “Multipotent Bone Marrow Stromal Cell*” OR “Adipose-Derived Mesenchymal Stromal Cells” OR “Adipose Derived Mesenchymal Stromal Cells” OR “Adipose Tissue-Derived Mesenchymal Stromal Cell*” OR “Adipose Tissue Derived Mesenchymal Stromal Cell*” OR “Mesenchymal Stromal Cell*” OR “Multipotent Mesenchymal Stromal Cell*” OR “Mesenchymal Progenitor Cell*”)

#5

TS=(“Neural Stem Cell*” OR “Induced Pluripotent Stem Cell*” OR “IPS Cell*” OR “hiPSC” OR “Adult Stem Cell*” OR “Somatic Stem Cell*” OR “Fetal Stem Cell*” OR “Embryonic Stem Cell*” OR “Multipotent Stem Cell*” OR “Totipotent Stem Cell*” OR “Hematopoietic Stem Cell*” OR “Hematopoietic Progenitor Cell*” OR “Hematopoietic Colony-Forming Unit*” OR “Peripheral Blood Stem Cell*” OR “Erythroid Precursor Cell*” OR “Erythroid Colony-Forming Unit*” OR “Erythroid Progenitor Cell*” OR “Erythroid Stem Cell*” OR “Erythropoietic Stem Cell*” OR “Erythropoietic Progenitor Cell*” OR “Erythroid Burst-Forming Unit*”)

**#6=#4 OR #5**

#7

TS=(“Exosome*” OR “extracellular vesicle*” OR “Secretory Vesicle*” OR “Cell-Derived Microparticle*” OR “secretome*” OR “membrane vesicle*” OR “Extracellular Vesicle*” OR “Exovesicle*” OR “Apoptotic Bod*”)

**#8=#3 AND #6 AND #7**

**3 Embase (529)**

#1

ischemic AND ('stroke'/exp OR stroke)

'ischemic stroke' OR 'ischaemic stroke' OR 'cryptogenic ischemic stroke' OR 'cryptogenic stroke' OR 'cryptogenic embolism stroke' OR 'wake-up stroke' OR 'wake up stroke' OR 'acute ischemic stroke'

#2

'cerebral infarction' OR 'cerebral infarct' OR 'left hemisphere, infarction, cerebral' OR 'infarction, left hemisphere, cerebral' OR 'cerebral, left hemisphere, infarction' OR 'infarction, cerebral, left hemisphere' OR 'subcortical infarction' OR 'infarction, subcortical' OR 'subcortical infarctions' OR 'posterior choroidal artery infarction' OR 'anterior choroidal artery infarction' OR 'infarction, right hemisphere, cerebral' OR 'right hemisphere, infarction, cerebral' OR 'cerebral, right hemisphere, infarction' OR 'infarction, cerebral, right hemisphere'

#3

'brain infarction' OR 'infarction, brain' OR 'brain infarct' OR 'infarct, brain' OR 'anterior circulation brain infarction' OR 'infarction, brain, anterior circulation' OR 'infarction, anterior circulation, brain' OR 'anterior circulation infarction, brain' OR 'venous infarction, brain' OR 'brain venous infarction' OR 'infarction, brain venous' OR 'venous infarctions, brain' OR 'infarction, venous brain' OR 'infarctions, venous brain' OR 'anterior cerebral circulation infarction' OR 'infarction, anterior cerebral circulation' OR 'posterior circulation infarction, brain' OR 'infarction, brain, posterior circulation' OR 'infarction, posterior circulation, brain'

**#4=#1 OR #2 OR #3**

#5

'stem cells' OR 'mesenchymal stem cells' OR 'neural stem cell' OR 'induced pluripotent stem cell' OR 'adult stem cell' OR 'fetal stem cell' OR 'embryonic stem cell' OR 'multipotent stem cell' OR 'totipotent stem cell' OR 'hematopoietic stem cell' OR 'peripheral blood stem cell' OR 'erythroid precursor cell' OR 'bone marrow stromal cell' OR 'multipotent bone marrow stromal cell' OR 'adipose-derived mesenchymal stromal cells' OR 'adipose derived mesenchymal stromal cells' OR 'adipose tissue-derived mesenchymal stromal cell' OR 'adipose tissue derived mesenchymal stromal cell' OR 'mesenchymal stromal cell' OR 'multipotent mesenchymal stromal cell' OR 'mesenchymal progenitor cell'

#6

'stem cell' OR 'progenitor cell' OR 'mother cell' OR 'colony-forming unit' OR 'colony forming unit' OR 'ips cell' OR 'hipsc' OR 'somatic stem cell' OR 'hematopoietic stem cell' OR 'hematopoietic progenitor cell' OR 'hematopoietic colony-forming unit' OR 'erythroid colony-forming unit' OR 'erythroid progenitor cell' OR 'erythroid stem cell' OR 'erythropoietic stem cell' OR 'erythropoietic progenitor cell' OR 'erythroid burst-forming unit'

**#7=#5 OR #6**

#8

'exosomes' OR 'extracellular vesicles' OR 'exosome' OR 'secretory vesicle' OR 'cell-derived microparticle' OR 'secretome' OR 'membrane vesicle' OR 'extracellular vesicle' OR 'exovesicle' OR 'apoptotic bodies'

**#9=#4 AND #7 AND #8**

**4 Cochrane Library (11)**

#1

MeSH：Ischemic Stroke

#2

Ischaemic Stroke or Stroke, Ischaemic or Ischaemic Strokes or Stroke, Ischemic or Ischemic Strokes or Wake up Stroke or Wake-up Strokes or Wake-up Stroke or Stroke, Wake-up or Cryptogenic Strokes or Cryptogenic Embolism Stroke or Stroke, Cryptogenic Embolism

#3

Stroke, Cryptogenic or Cryptogenic Embolism Strokes or Ischemic Stroke, Cryptogenic or Cryptogenic Ischemic Strokes or Cryptogenic Stroke or Stroke, Cryptogenic Ischemic or Embolism Stroke, Cryptogenic or Cryptogenic Ischemic Stroke or Acute Ischemic Stroke or Stroke, Acute Ischemic or Acute Ischemic Strokes or Ischemic Stroke, Acute

#4

MeSH：Cerebral Infarction

#5

Posterior Choroidal Artery Infarction or Infarctions, Cerebral or Cerebral Infarctions or Cerebral Infarct or Infarction, Cerebral or Cerebral Infarcts or Infarct, Cerebral or Infarcts, Cerebral or Subcortical Infarction or Subcortical Infarctions or Infarctions, Subcortical or Infarction, Subcortical or Anterior Choroidal Artery Infarction or Cerebral Infarction, Right Hemisphere or Cerebral, Right Hemisphere, Infarction

#6

Infarction, Right Hemisphere, Cerebral or Right Hemisphere, Infarction, Cerebral or Right Hemisphere, Cerebral Infarction or Infarction, Cerebral, Right Hemisphere or Cerebral Infarction, Left Hemisphere or Left Hemisphere, Infarction, Cerebral or Left Hemisphere, Cerebral Infarction or Infarction, Cerebral, Left Hemisphere or Infarction, Left Hemisphere, Cerebral or Cerebral, Left Hemisphere, Infarction

#7

MeSH：Brain Infarction

#8

Brain Infarction, Anterior Circulation or Infarction, Anterior Circulation, Brain or Infarction, Brain, Anterior Circulation or Anterior Circulation Brain Infarction or Infarctions, Brain or Brain Infarct or Brain Infarcts or Infarction, Brain or Infarct, Brain or Brain Infarctions or Infarcts, Brain or Infarction, Brain, Posterior Circulation or Infarction, Posterior Circulation, Brain or Posterior Circulation Infarction, Brain or Posterior Circulation Brain Infarction or Brain Infarction, Posterior Circulation

#9

Brain Venous Infarctions or Venous Brain Infarctions or Brain Infarctions, Venous or Infarctions, Venous Brain or Venous Infarctions, Brain or Brain Venous Infarction or Venous Brain Infarction or Brain Infarction, Venous or Infarction, Brain Venous or Venous Infarction, Brain or Infarctions, Brain Venous or Infarction, Venous Brain or Infarction, Anterior Cerebral Circulation or Anterior Cerebral Circulation Infarction or Anterior Circulation Infarction, Brain

**#10= #1 or #2 or #3 or #4 or #5 or #6 or #7 or #8 or #9**

#11

MeSH：Exosomes

#12

MeSH：Extracellular Vesicles

#13

Exovesicles or Exovesicle or Apoptotic Body or Bodies, Apoptotic or Body, Apoptotic or Apoptotic Bodies or Vesicle, Extracellular or Vesicles, Extracellular or Extracellular Vesicle

**#14= #11 or #12 or #13**

**#15=#10 and #14**

**5 Scopus (301)**

(( TITLE-ABS-KEY ( "ischemic stroke" OR "ischemic stroke" OR "ischaemic stroke*" OR "cryptogenic ischemic stroke*" OR "cryptogenic stroke*" OR "cryptogenic embolism stroke*" OR "wake-up stroke*" OR "wake up stroke*" OR "acute ischemic stroke*" ) ) OR ( TITLE-ABS-KEY ( "cerebral infarction" OR "cerebral infarct*" OR "left hemisphere, infarction, cerebral" OR "infarction, left hemisphere, cerebral" OR "cerebral, left hemisphere, infarction" OR "infarction, cerebral, left hemisphere" OR "subcortical infarction" OR "infarction, subcortical" OR "subcortical infarctions" OR "posterior choroidal artery infarction" OR "anterior choroidal artery infarction" OR "infarction, right hemisphere, cerebral" OR "right hemisphere, infarction, cerebral" OR "cerebral, right hemisphere, infarction" OR "infarction, cerebral, right hemisphere" ) ) OR ( TITLE-ABS-KEY ( "brain infarction" OR "infarction, brain" OR "brain infarct*" OR "infarct, brain" OR "anterior circulation brain infarction" OR "infarction, brain, anterior circulation" OR "infarction, anterior circulation, brain" OR "anterior circulation infarction, brain" OR "venous infarction, brain" OR "brain venous infarction*" OR "infarction, brain venous" OR "venous infarctions, brain" OR "infarction, venous brain" OR "infarctions, venous brain" OR "anterior cerebral circulation infarction" OR "infarction, anterior cerebral circulation" OR "posterior circulation infarction, brain" OR "infarction, brain, posterior circulation" OR "infarction, posterior circulation, brain" ) )) **AND** (( TITLE-ABS-KEY ( "stem cell*" OR "progenitor cell*" OR "mother cell*" OR "mesenchymal stem cells" OR "bone marrow stromal cell*" OR "multipotent bone marrow stromal cell*" OR "adipose-derived mesenchymal stromal cells" OR "adipose derived mesenchymal stromal cells" OR "adipose tissue-derived mesenchymal stromal cell*" OR "adipose tissue derived mesenchymal stromal cell*" OR "mesenchymal stromal cell*" OR "multipotent mesenchymal stromal cell*" OR "mesenchymal progenitor cell*" ) ) OR ( TITLE-ABS-KEY ( "neural stem cell*" OR "induced pluripotent stem cell*" OR "ips cell*" OR "hipsc" OR "adult stem cell*" OR "somatic stem cell*" OR "fetal stem cell*" OR "embryonic stem cell*" OR "multipotent stem cell*" OR "totipotent stem cell*" OR "hematopoietic stem cell*" OR "hematopoietic progenitor cell*" OR "hematopoietic colony-forming unit*" OR "peripheral blood stem cell*" OR "erythroid precursor cell*" OR "erythroid colony-forming unit*" OR "erythroid progenitor cell*" OR "erythroid stem cell*" OR "erythropoietic stem cell*" OR "erythropoietic progenitor cell*" OR "erythroid burst-forming unit*" ) )) **AND** (TITLE-ABS-KEY ( "exosome*" OR "extracellular vesicle*" OR "secretory vesicle*" OR "cell-derived microparticle*" OR "secretome*" OR "membrane vesicle*" OR "extracellular vesicle*" OR "exovesicle*" OR "apoptotic bod*" ))
